# Multi-Trait GWAS and New Candidate Genes Annotation for Growth Curve Parameters in Brahman Cattle

**DOI:** 10.1371/journal.pone.0139906

**Published:** 2015-10-07

**Authors:** Aline Camporez Crispim, Matthew John Kelly, Simone Eliza Facioni Guimarães, Fabyano Fonseca e Silva, Marina Rufino Salinas Fortes, Raphael Rocha Wenceslau, Stephen Moore

**Affiliations:** 1 Department of Animal Science, Universidade Federal de Viçosa, Viçosa, Minas Gerais, Brazil; 2 Queensland Alliance for Agriculture & Food Innovation University of Queensland, Brisbane, Queensland, Australia; 3 School of Chemistry and Molecular Bioscience, the University of Queensland, Brisbane, Queensland, Australia; 4 Animal Science Institute, Universidade Federal de Minas Gerais, Belo Horizonte, Minas Gerais, Brazil; Pennsylvania State University, UNITED STATES

## Abstract

Understanding the genetic architecture of beef cattle growth cannot be limited simply to the genome-wide association study (GWAS) for body weight at any specific ages, but should be extended to a more general purpose by considering the whole growth trajectory over time using a growth curve approach. For such an approach, the parameters that are used to describe growth curves were treated as phenotypes under a GWAS model. Data from 1,255 Brahman cattle that were weighed at birth, 6, 12, 15, 18, and 24 months of age were analyzed. Parameter estimates, such as mature weight (A) and maturity rate (K) from nonlinear models are utilized as substitutes for the original body weights for the GWAS analysis. We chose the best nonlinear model to describe the weight-age data, and the estimated parameters were used as phenotypes in a multi-trait GWAS. Our aims were to identify and characterize associated SNP markers to indicate SNP-derived candidate genes and annotate their function as related to growth processes in beef cattle. The Brody model presented the best goodness of fit, and the heritability values for the parameter estimates for mature weight (A) and maturity rate (K) were 0.23 and 0.32, respectively, proving that these traits can be a feasible alternative when the objective is to change the shape of growth curves within genetic improvement programs. The genetic correlation between A and K was -0.84, indicating that animals with lower mature body weights reached that weight at younger ages. One hundred and sixty seven (167) and two hundred and sixty two (262) significant SNPs were associated with A and K, respectively. The annotated genes closest to the most significant SNPs for A had direct biological functions related to muscle development (*RAB28*), myogenic induction (*BTG1*), fetal growth (*IL2*), and body weights (*APEX2*); K genes were functionally associated with body weight, body height, average daily gain (*TMEM18*), and skeletal muscle development (*SMN1*). Candidate genes emerging from this GWAS may inform the search for causative mutations that could underpin genomic breeding for improved growth rates.

## Introduction

In beef cattle, postnatal body weight is often recorded repeatedly at different ages for the same individual and is a typical example of longitudinal data which the trait of interest changes gradually and continually over time. The term growth curve is used as a general designation for such data, and reflects the lifetime interrelationships between an individual's inherent potential to grow and mature in all body parts[1].There are different models to describe growth curves in livestock animals[2], and these models allow us to summarize the weight-age gain through a few parameters, such as mature weight (A) and maturity rate (K),which explain the whole growth process under a biological scenario. When fitting these different models to a particular dataset, the use of goodness of fit measures are needed to choose the best model to describe the growth curve to the population in question.

Considering a specific model, individual animal growth curves might be assessed and differences between individuals may partly reflect the genetic impacts on body weight, with some genes contributing at different levels to the overall growth trajectory. Historically, changes in the shape of growth curves have been assessed by using a quantitative genetic knowledge[1,3–5]assuming the parameters (commonly A and K) estimates as phenotypes under a mixed model approach. In relation to QTLs affecting growth curves, the theory of functional mapping proposed by Ma C-X et al.[6] is quite general and can be applied to any dynamic complex traits [7], such as beef cattle body weights over time. Although these studies have been the basis of QTL detection for longitudinal traits, the postulated theory is based on linkage analysis by using highly spaced markers (low density) in specific designed population mapping.

In the current post-genomic era, genome-wide association studies (GWAS) based on high density markers could demand new strategies of QTL mapping, even in non-specific designed populations. In this context, Das et al. [8] proposed a method based on random regression enabling them to exploit, among several points, non-additive effects and specific covariance functions to describe changes in the SNP effect markers over time. Das et al.[9] generalized this method to a multi-trait approach, making it even more powerful and applicable to situations involving more than one trait. Even though these random regression-based models are currently the most sophisticate and flexible tools to assess GWAS for longitudinal traits, it does not directly provide biological-interpretable parameters such as mature weight (A) and maturity rate (K), which are usually required in animal breeding programs.

Although representing an obvious way to access the genetic underpinning growth curve changes, the use of molecular markers associated with growth curve parameters was attempted herein for the very first time. Current studies have limited the understanding of the genetic architecture of beef cattle growth to simple QTL detection from GWAS by using body weight at specific ages as phenotypes[10,11]. Further understanding of beef cattle growth must be extended by considering the whole growth trajectory over time under a growth curve GWAS approach.

Since the seminal study of Fitzhugh[1] about growth curves in the context of animal breeding, the genetic correlations between the parameter (mainly A and K) estimates have been considered in scientific studies. These genetic correlations could be attributed to quantitative trait loci (QTL) that have pleiotropic effects on multiple parameters or to closely linked QTL, each affecting different parameters. In this context, a multi-trait GWAS might help to explain these correlations. Multi-trait methods have already been successfully used to identify QTL sustaining genetic correlations in beef cattle, such as growth and intake components of feed efficiency[12]as well as stature, fatness, and reproduction[13,14]. However, studies still carried the above mentioned limitation: phenotypes were original measurements at a given age or condition. To our knowledge, there is no reports of multi-trait GWAS applied to growth curve parameters. Thus, we believe that the present study can be a solid reference for future researchers interested in applying GWAS to other longitudinal traits of economic importance in animal production, such as lactation and egg production curves.

It is expected that the integration of statistical modeling (growth curve GWAS) and functional genomics (gene function annotation and ontology analyses) may be useful for deciphering the genetic mechanisms sustaining individual variation of growth curves in beef cattle. Functional genomics have been applied to reproductive traits in cattle, revealing candidate genes and molecular markers that proved useful for genomic selection [15]. Candidate genes were associated with growth traits in cattle such as bodyweight, height, or average daily gain; these include, for example,*PLAG1*,*PDE4B*, *LEPR*, *CYP2J2*, and *FGGY*[16–18]. However, these candidate genes may not reflect the functional genomics of growth curve parameters, which data are unknown.

In this study, we compared and chose the best fitting nonlinear model from five models describing the weight-age data of a Brahman cattle population. Our major aim was to use the estimated parameters from the best model as phenotypes in a multi-trait GWAS and identify and characterize single nucleotide polymorphisms (SNPs) associated with the parameters. Once SNPs were associated, we progressed to indicate SNP-derived candidate genes and to discuss their annotated functions with relation to the biological processes of growth in beef cattle.

## Material and Methods

Animal Care and Use Committee approval was not required for this study because the data were obtained from pre-existing phenotypic databases and DNA storage sets. Data used herein were gathered by the Cooperative Research Centre for Beef Genetic Technologies (Beef CRC). Experimental design details and general information regarding the Beef CRC cattle herd was published elsewhere[19].

### Phenotypic and genotypic data

Data were comprised of six records of body weight from Brahman cattle, which were born between 2004 and 2010. A total of 1,642 animals had at least one measurement of body weight, but1,255 animals were weighed at birth, and at the sixth, twelfth, fifteenth, eighteenth, and twenty-fourth months of age, therefore comprising 7,530 records of body weights. From the broad beef CRC population, we only included animals with complete records (six weight-age data points).

The original body weight measurements at each age were pre-adjusted for fixed effects (contemporary group, year/month/birthday) by using a linear model fitted separately for the data from each age. This methodology is generally used in analysis that consider nonlinear models parameters estimates (e.g. mature weight, A, and maturity rate, K) as phenotypes in mixed models, because these effects are related to the observed body weights instead of the growth curve parameter estimates. Differently, the use of linear random regression models (RRM), such as Legendre polynomials and splines, allows the inclusion of different effects in the same model. On the other hand, RRM do not provide biological interpretation to the estimated coefficients such as A and K from nonlinear regression.

The summary containing the descriptive statistics of the pre-adjusted phenotypic data is presented in Table 1.

**Table 1:** Means, standard deviations (SD), and ranges for body weight (kg) measurements at six different ages of 1,255 Brahman cattle.

Age (months)	Mean weight (SD)	Min	Max
0	35.3(5.26)	17.47	55.5
6	203.6 (23.87)	109.2	298
12	247.0(24.43)	160.7	334.1
15	297.8 (27.5)	188.4	397.5
18	353.4(29.38)	212.7	471.5
24	384.4(34.31)	210.5	505.4

Genotyping was performed by using the BovineHD BeadChip of Illumina (San Diego, CA, USA) and by using the UMD 3.1 assembly of the bovine genome sequence for the mapping information. A total of 1,255 samples were genotyped for 777,000 SNPs, and the quality control (QC) analysis was applied to this genotype data. Individuals with call rates <95% and markers with call rates <95% and/or minor allele frequency (MAF) <0.01 were excluded in each population. Markers that deviated from the Hardy-Weinberg equilibrium (HWE) (P <10^−7^) were also removed. After the QC analysis, a total of 729,068 markers were considered. There were 941 genotyped animals with 6 records of body weight each. Since the GWAS was based on a polygenic mixed model approach, the pedigree file that was used to calculate the relationship matrix was composed of 17,021 individuals.

### Growth curve fitting

Five of the most widely used nonlinear models (Table 2) to describe animal growth curves, Brody[20], Logistic[21], von Bertalanffy [22], Gompertz [23], and Richards [24],were fitted for each animal by using the iterative nonlinear least squares method via the Gauss-Newton algorithm implemented in the package *nlme* (Nonlinear Mixed-Effects Model) of R software[25].

**Table 2:** Nonlinear regression models fitted to growth curve data (weight-age) of Brahman cattle.

Model	Function	Number of parameters
Brody	w_t_ = A(1-bexp^-Kt^)	3
von Bertalanffy	w_t_ = A(1-bexp^-Kt^)^3^	3
Logistic	w_t_ = A(1+bexp^-Kt^)^-1^	3
Gompertz	w_t_ = Aexp(-bexp^-Kt^)	3
Richards	w_t_ = A(1±bexp^-Kt^)^M^	4

w_t_: body weight at ages t = 0, 6, 12, 15, 18, and 24 months; A: asymptotic or mature weight; b: parameter of integration (or the time-scale parameter); K: maturity rate; M: inflection parameter of Richards function.

In general, the growth curve parameters (Table 2) may be interpreted as follows [1]:A represents the mature body weight (adult or asymptotic weight), maintained independently of short-term fluctuations; b is the integration parameter, and indicates the proportion of the asymptotic mature weight to be gained after birth (has no relevant biological interpretation); k is the maturity rate (growth precocity measure), representing the rate of approach to the mature body weight.

In order to determine the nonlinear model that best describes the growth curve of the studied Brahman cattle population, the following goodness of fit measures were used: adjusted coefficient of determination (R^2^), mean square error (MSE), convergence rate (C%), Akaike`s Information Criterion (AIC), and mean absolute deviation (MAD) in two periods of the curve (MAD_1_ e MAD_2_). MAD evaluates the model`s fitting according to the difference between predicted and observed body weights in different parts of growth curve[26]. In the present study, it was performed to evaluate the ability to provide consistent predictions for body weight in the first three measures, that comprises birth, 6, and 12 months (MAD_1_); and15, 18, and 24 months (MAD_2_). Since we used the *nlme* function of R software, that is based on nonlinear mixed models, the adjusted coefficient of determination (R^2^), mean square error (MSE), mean-absolute deviation for periods one (MAD_1_) and two (MAD_2_) were calculated for each animal taking into account the individual parameter estimates (A, b and K) postulated as deviations of overall growth curve (population curve). In this context, the AIC values were calculated separately for each model, since all animals were considered jointly in the nonlinear mixed model fitting.

The AIC and BIC criteria were also used to compare all presented growth models considering different correlation matrices (corAR1, corCAR1 and corARMA1) aiming to point out for the best within-individual correlation structure. The mentioned matrices were implemented by using the *corr* statement of *nlme* function from R software (S1 Script).

### Genome-wide association studies

After selecting the nonlinear model that best fit the body weight data, only animals with phenotypes and genotypes were used in genome-wide association studies (GWAS), that was performed for all three traits (A, b, and K parameters estimates); this was accomplished by using a multiple trait analysis in order to test the association of a given marker simultaneously with these three traits. In order to point out for some pre-existent sub-populations (correction for family structure effects), the polygenic random effect was added to the model according to Goddard and Hayes[27]. Thus, the following multi-trait mixed model was considered:

Y=Ws+Zu+e, (1)

where **Y** represents the vector of observations from the three phenotypes (A, b, and K estimates) for each animal; **s** represents the SNP effects vector (one value for each trait); **u** is the vector of random animal polygenic effect, **u** ∼ N(**0**, ∑_u_ ⊗**A**), where **A** is the additive pedigree relationship matrix, and **e** represents the residual vector, **e** ∼ N(**0**, ∑_e_ ⊗**I**). The matrices **W** and **Z** represent the incidence matrices for **s** and **u**, respectively. Besides the estimated SNP effects, this model also allows for estimation of the heritabilities and genetic correlations by using the estimates of the genetic and residual covariance matrices (∑^u and ∑^e), as well as the prediction of the genetic breeding value ($\hat{u}$). These covariance matrices are provided by the following:

$\Sigma_{u}=\left[\begin{array}{ccc} \sigma_{A_{u}}^{2} & \sigma_{A_{u}}{}_{b_{u}} & \sigma_{A_{u}}{}_{K_{u}}\\ \; & \sigma_{b_{u}}^{2} & \sigma_{b_{u}}{}_{K_{u}}\\ sim & \; & \sigma_{K_{u}}^{2} \end{array}\right]$ and $\Sigma_{e}=\left[\begin{array}{ccc} \sigma_{A_{e}}^{2} & \sigma_{A_{e}}{}_{b_{e}} & \sigma_{A_{e}}{}_{K_{e}}\\ \; & \sigma_{b_{e}}^{2} & \sigma_{b_{e}}{}_{K_{e}}\\ sim & \; & \sigma_{K_{e}}^{2} \end{array}\right]$, thus enabling the estimation of relevant genetic parameters, such as heritabilities and genetic correlations. In general terms, the model presented in (1) can be rewritten as follow:

$$\left[\begin{array}{c} Y_{\text{A}}\\ Y_{\text{b}}\\ Y_{\text{K}} \end{array}\right]=\left[\begin{array}{ccc} W_{\text{A}} & 0 & 0\\ 0 & W_{\text{b}} & 0\\ 0 & 0 & W_{\text{K}} \end{array}\right]\left[\begin{array}{c} {\text{s}}_{\text{A}}\\ {\text{s}}_{\text{b}}\\ {\text{s}}_{\text{K}} \end{array}\right]+\left[\begin{array}{ccc} Z_{\text{A}} & 0 & 0\\ 0 & Z_{\text{b}} & 0\\ 0 & 0 & Z_{\text{K}} \end{array}\right]\left[\begin{array}{c} u_{\text{A}}\\ u_{\text{b}}\\ u_{\text{K}} \end{array}\right]+\left[\begin{array}{c} e_{\text{A}}\\ e_{\text{b}}\\ e_{\text{K}} \end{array}\right],$$

where each sub-matrix **W** represents the genotypes of each SNP, which was coded as 0 for the homozygote of the first allele, 1 for the heterozygote, and 2 for the homozygote of the second allele.

Under a GWAS approach, this model was fitted individually for each SNP, where the output is a vector of marker effect estimates for each trait, i.e. $\hat{s}=\left[{\hat{\text{s}}}_{\text{A}},{\hat{\text{s}}}_{\text{b}},{\hat{\text{s}}}_{\text{k}}\right]'$. These SNP effects were ranked according to the significance of association with each trait and the cutoff for considering a significant association was established by Benjamini and Hochberg multiple testing correction of the P-value (false discovery rate < 0.05;[28]) adopting a P-value threshold of 0.001.

In addition to the marker effect estimates, the percentage of the genetic variance accounted by the i^th^ SNP (V_i_) was also estimated in order to facilitate the identification of possible relevant chromosome regions related to growth curve parameters. The following equation was used to estimate this percentage per SNP:

$${\text{V}}_{\text{i}}=100\times \frac{{2\text{p}}_{\text{i}}{\text{q}}_{\text{i}}{\hat{\text{s}}}_{\text{i}}^{2}}{\sum_{\text{i}=1}^{729,068}{2\text{p}}_{\text{i}}{\text{q}}_{\text{i}}{\hat{\text{s}}}_{\text{i}}^{2}},$$

where p_i_ and q_i_ are the allele frequencies for the i^th^ SNP estimated across the entire population and ${\hat{\text{s}}}_{\text{i}}^{2}$ is the estimated additive effect (from model 1)of the i^th^ SNP squared. Since the percentage of the variance explained by a specific marker may be very small (mainly when using BovineHD BeadChip), and very often without practical interpretation, we adopted a haplotype block analysis in order to group related markers aiming to increase the variance explained by this group. To achieve this, the software Haploview 4.0 [29]was used to examine measures of linkage disequilibrium (r^*2*^) between adjacent SNPs and to define haplotype block structures based on the definition by Gabriel et al [30].The variance explained by a given haplotype block containing N markers can be determined by $\sum_{\text{i}=1}^{\text{N}}{\text{V}}_{\text{i}}$.

Fixed effects were not considered at this step, since they were already taken into account before fitting nonlinear functions (see pre-adjustment for fixed effects in the topic “phenotypic and genotypic data”). Solutions to the effects in the model (1) as well as variance components were estimated by using WOMBAT software[31]. The corrected p-value (FDR) were log transformed for visualization in Manhattan plots, which were built by using the *mhtplot* function of R software [25].

We also evaluated the performance of the multi-trait GWAS mixed model by using simulated data, which was provided by QTLMAS2009 and fully described in Coster et al [32]. It consists of 100 full-sib families, each with 20 offspring. Half of the offspring have both phenotype information of yield at 5 distinct time points (0, 132, 25, 397, 530) and genotype data from 453 SNP markers distributed over 5 chromosomes of 1 Morgan each. Phenotypes were simulated according to a logistic growth curve and were made available for 1,000 offspring individuals.

In order to validate the simulation study, we performed ten replicates using permutations from the individuals’ records over time, resulting in new data sets. For this, it was ensured that the records were shuffled separately for each time point, which means that a given individual received randomly only records from other individuals measured in the same time point. This permutation constraint was essential to guarantee the behavior of the original longitudinal pattern (sigmoid growth trajectory).A logistic nonlinear growth model was fitted to the records over time generating ϕ_1_, ϕ_2_ and ϕ_3_parameter estimates of each individual. Hereafter, multi-trait GWAS mixed model was applied to QTL detection, considering ϕ_1_, ϕ_2_ and ϕ_3_ estimates as phenotypes. Each parameter was influenced by six QTLs (one QTL had a large effect and five QTLs had small effects), located on five evaluated chromosomes. The results were computed in terms of percentage of detected true QTLs in ten replicates.

### Gene function annotation

We exploited the biological mechanism of growth underlying the significant SNPs based on the interpretability of the gene functions related to the relevant SNPs. In order to track the genes with markers inside or close to markers, we used the package Map2NCBI [33] of R software based on the UMD Bos taurus 3.1 assembly of the bovine genome sequence.

To provide information regarding the identity and function of genes at mapped SNP markers, the chromosomal positions at the Ensembl Genome Browser[34]were used. Lists of genes located nearest to the significant SNP were extracted, allowing for a maximum distance of 1 Mb between SNP and annotated genes. Putative genes identified for Brahman breeds were established by a BLAST Homology search of known, identified human gene transcripts, which were downloaded from the genome databanks National Center for Biotechnology Information (NCBI)[35]. The biological function of these genes and their possible relation to growth traits were investigated, and when no information was available for the *Bos taurus* genes, human, rat, and mouse biological function annotations were used to proceed with the *in-silico* functional analyses. Animal QTLdb[36]was accessed to verify previous QTL that were reported for growth traits in the surrounding regions of our significant SNPs. Thus, it was possible to identify the biological mechanisms and functions involving the identified genes as well as highlight the most relevant of them that are putatively associated with growth curves in Brahman cattle.

## Results

### Growth curve fitting and estimated genetic parameters

The summary of growth curve parameter estimates for all models, as well as the goodness of fit analysis that was used to reveal the most appropriate model to describe the growth curve of Brahman cattle is given in Table 3.

**Table 3:** Means and standard deviations for the parameter estimates, and goodness of fit(GOF) results based on convergence rate (C%), adjusted coefficient of determination (R^2^); mean square error (MSE); mean-absolute deviation for periods 1 (until 12 months–MAD_1_) and 2 (older than 12 months–MAD_2_), and Akaike's information criterion (AIC).

Parameter	Models			
	**Gompertz**	**Logistic**	**Brody**	**von Bertalanffy**
A	419.90(51.44)	399.85(42.67)	520.32(163.72)	373.86(36.26)
b	2.00(0.12)	4.85(0.64)	0.92(0.02)	0.75(0.04)
K	0.13(0.03)	0.20(0.044)	0.06(0.02)	0.08(0.02)
**GOF**	**Gompertz**	**Logistic**	**Brody**	**von Bertalanffy**
C%	100	99.44	99.92	99.52
R^2^	0.96(0.01)	0.95(0.02)	0.98(0.01)	0.87(0.03)
MSE	604.91(224.70)	821.16(273.40)	382.79(167.01)	2,071.96(618.33)
MAD_1_	27.53(5.81)	32.83(6.18)	18.94(5.32)	46.61(10.70)
MAD_2_	10.95(3.72)	10.51(4.31)	10.28(3.34)	11.87(6.06)
AIC	42.90(2.39)	44.81(2.18)	40.01(2.76)	50.42(2.033)

We compared the models considered in Table 3 assuming special within-individual correlation structures (corAR1, corCAR1 and corARMA1). Other more complex correlation structures were also considered, but they presented convergence problems. The goodness of fit analysis based on Akaike Information Criterion (AIC) revealed the superiority (S4 Table) of the simplest model (assuming independence), maybe because the pre-adjustment of the phenotypic data for fixed effects might have dispelled some possible correlation between observations over time. Thus, results presented in Table 3 reflect the goodness of fit analysis for different nonlinear growth models considering the best identified correlation structure.

All tested models had high convergence rates (Table 3), which were very close to 100%; therefore, this information could not be used to discriminate the alternative growth curve models (Table 3). The Richards model did not achieve the convergence for the majority of animals (C%<10); thus, it was excluded from this study, as there is no practical reason to consider a model presenting a low convergence rate. The Brody model presented with a higher coefficient of determination (R^2^), the lowest mean squares error (MSE),the lowest mean absolute deviation for both initial and final parts of the curve (MAD_1_ and MAD_2_), and the lowest Akaike's Information Criterion (AIC). Thus, it was chosen as the most appropriate to describe the growth curve of Brahman cattle in the present study.

The heritability values of 0.23, 0.41, and 0.31 for A, b, and K respectively indicate that these traits can be a feasible alternative for breeding programs, when the aim is to produce efficient animals considering the growth curve. The genetic correlation between A and K was -0.84, between A and b was 0.78, and between K and b it was -0.88. Thus, direct selection of a higher mature weight (A) leads to animals take longer to reach maturity (result of a lower maturity rate–K).

### Genome-wide association studies

**Fig 1:**
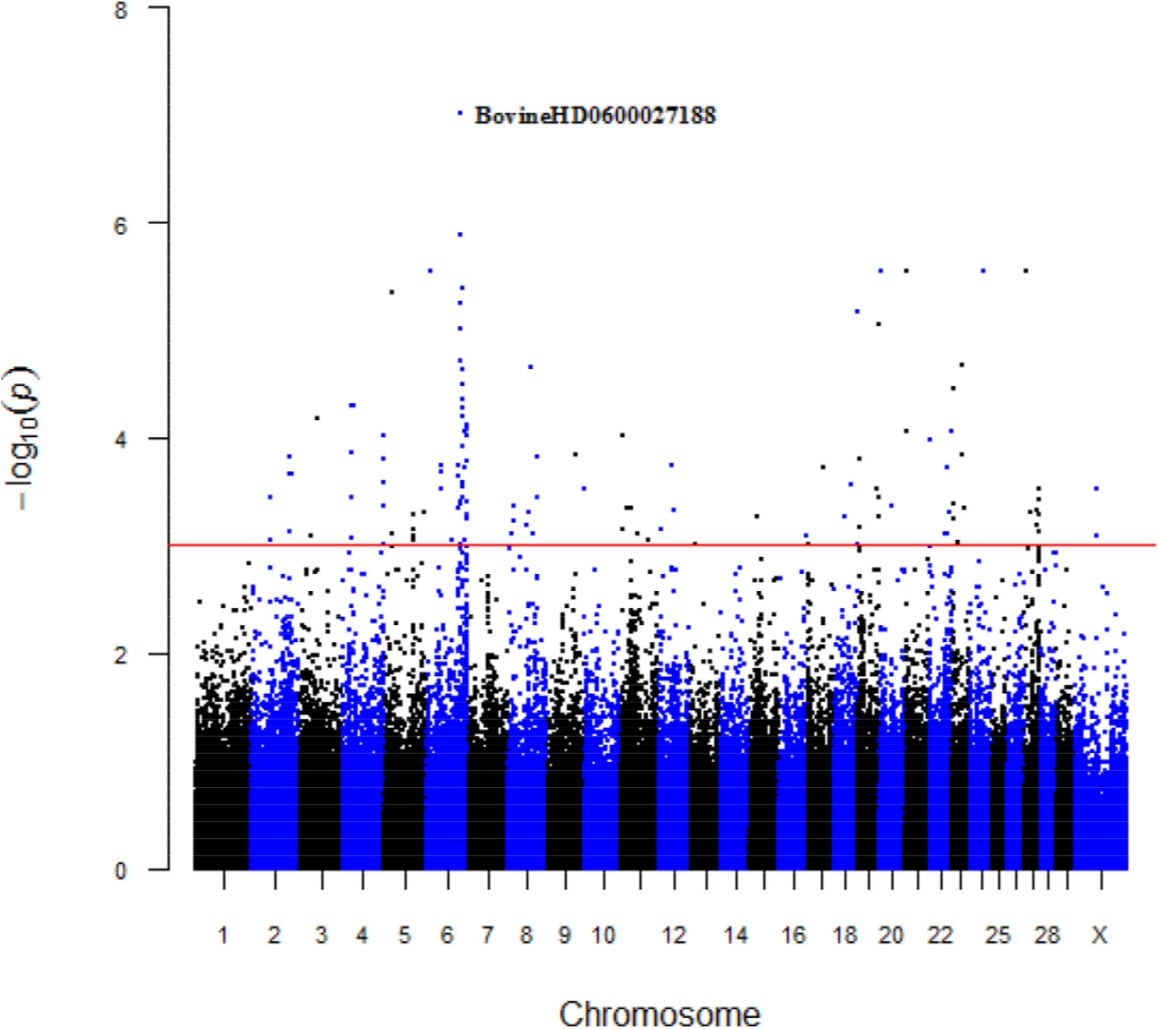
Manhattan plots for the growth curve parameter mature weight (A) in Brahman cattle. Chromosomes 1 to 29 and X are shown, separated by alternating colors. The corresponding horizontal lines indicate the genome-wide significance levels for both traits.

**Fig 2:**
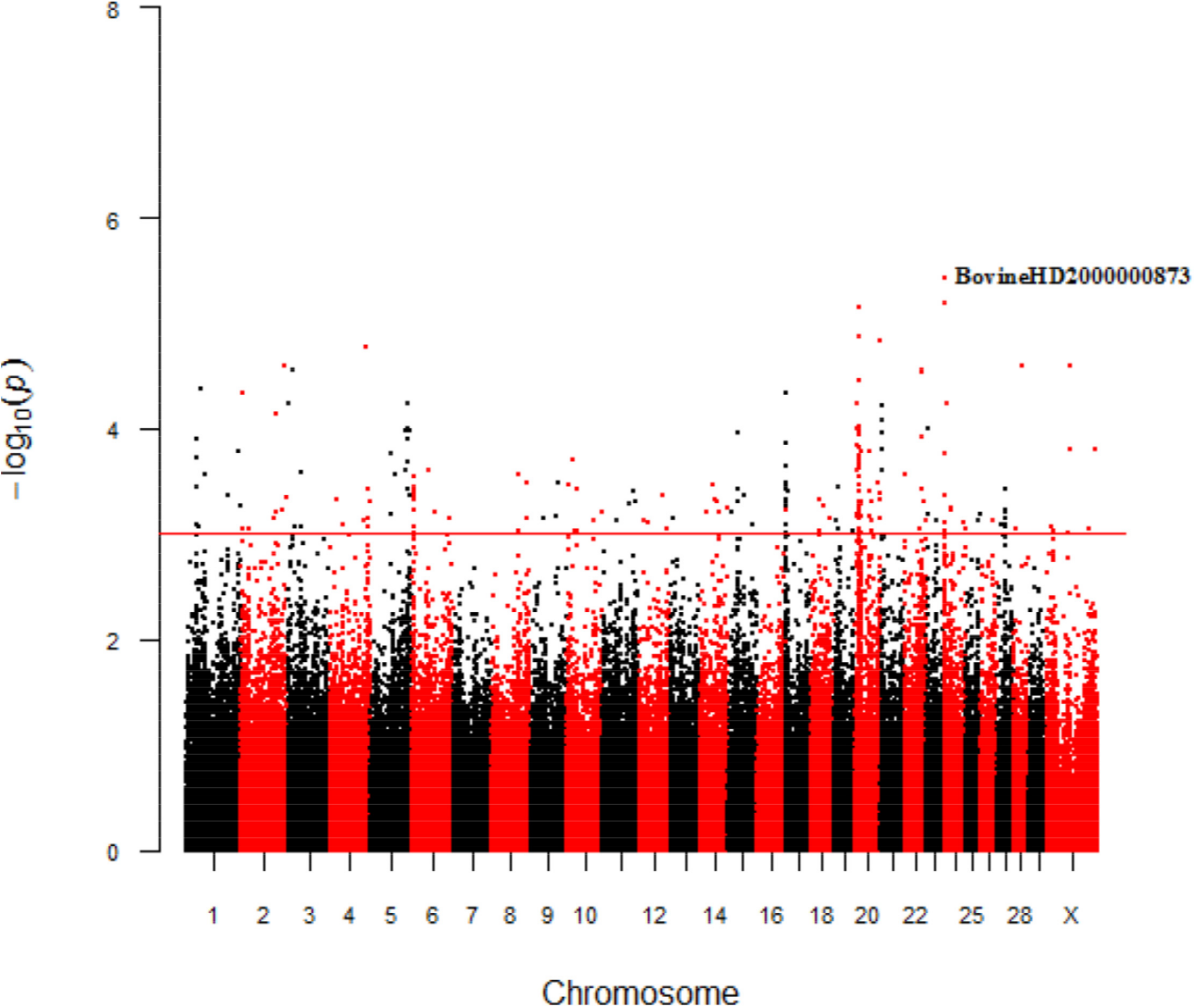
Manhattan plots for the growth curve parameter maturity rate (K) in Brahman cattle. Chromosomes 1 to 29 and X are shown, separated by alternating colors. The corresponding horizontal lines indicate the genome-wide significance levels for both traits.

The Manhattan plots pointed 167 and 262 significant SNPs associated with mature weight (A) and maturity rate (K), respectively (Fig 1 and Fig 2). The peak that was observed for A was composed of 44 significant SNPs, which started from 13417489 to 114954346 bp on BTA6, and the SNP with the lowest p-value was located at 97907606 bp. In spite of K, Manhattan plots indicated 64 significant SNPs at BTA20, starting from 1876553 to 69791421 bp, and the SNP with the lowest p value was located at 11089453 bp.

The majority of SNPs with lower p-values are located on chromosomes 4, 5, 6, 8, and 27 for trait A (mature weight), and chromosomes 5, 20, and 24 for trait K (maturity rate). For trait A, around 25% of the significant SNPs were located on chromosome 6, where A was observed (Fig 1), and those SNPs were related to 12 genes (S1 Table).

For trait K, the Manhattan plot revealed that almost 25% of significant SNPs are on chromosome 20 (Fig 2), and these markers were associated with 10 genes (S2 Table).

**Fig 3:**
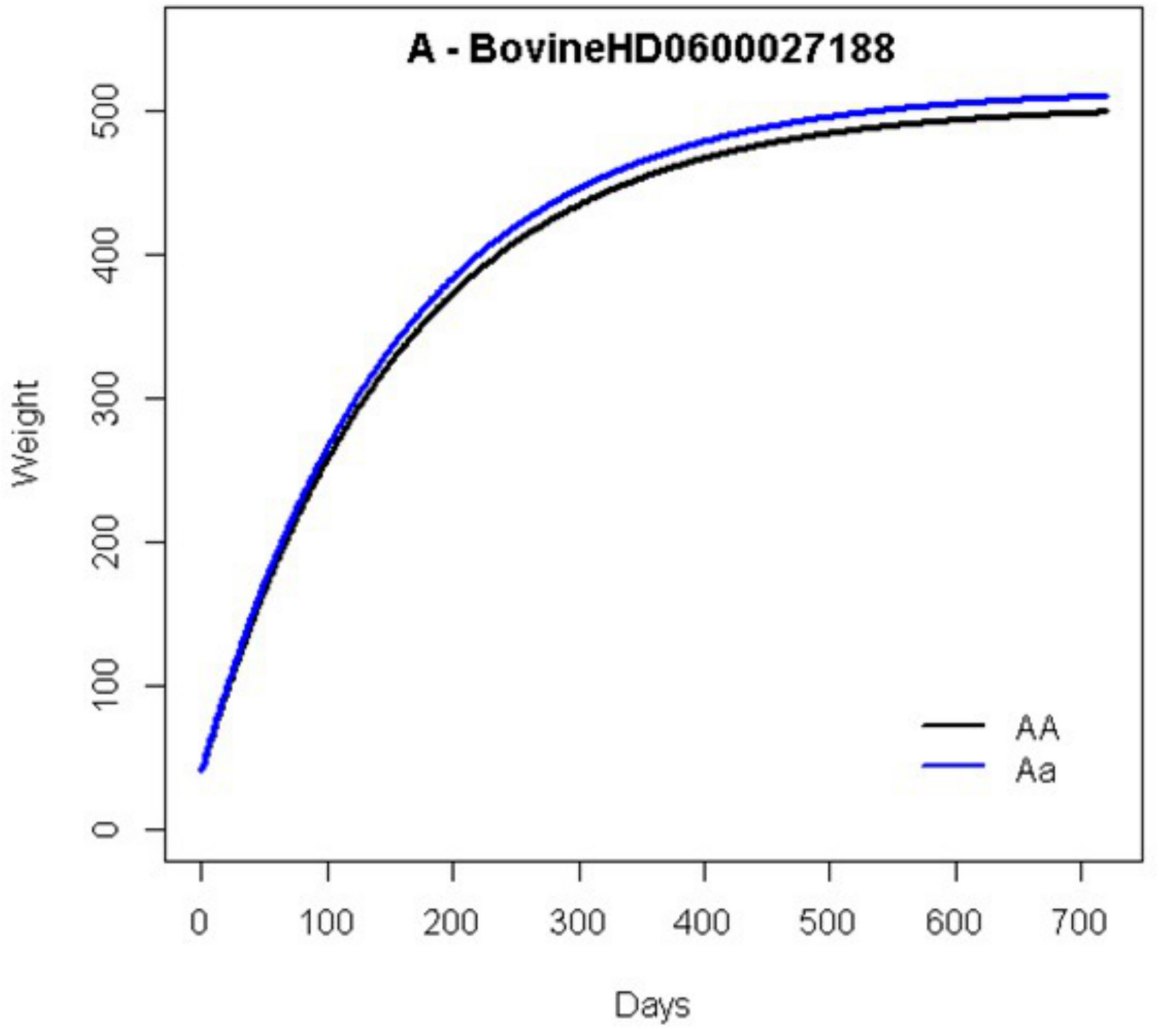
Estimated growth curves based on the Brody nonlinear model for genotypes of the most significant SNPs for mature weight (BovineHD0600027188).

**Fig 4:**
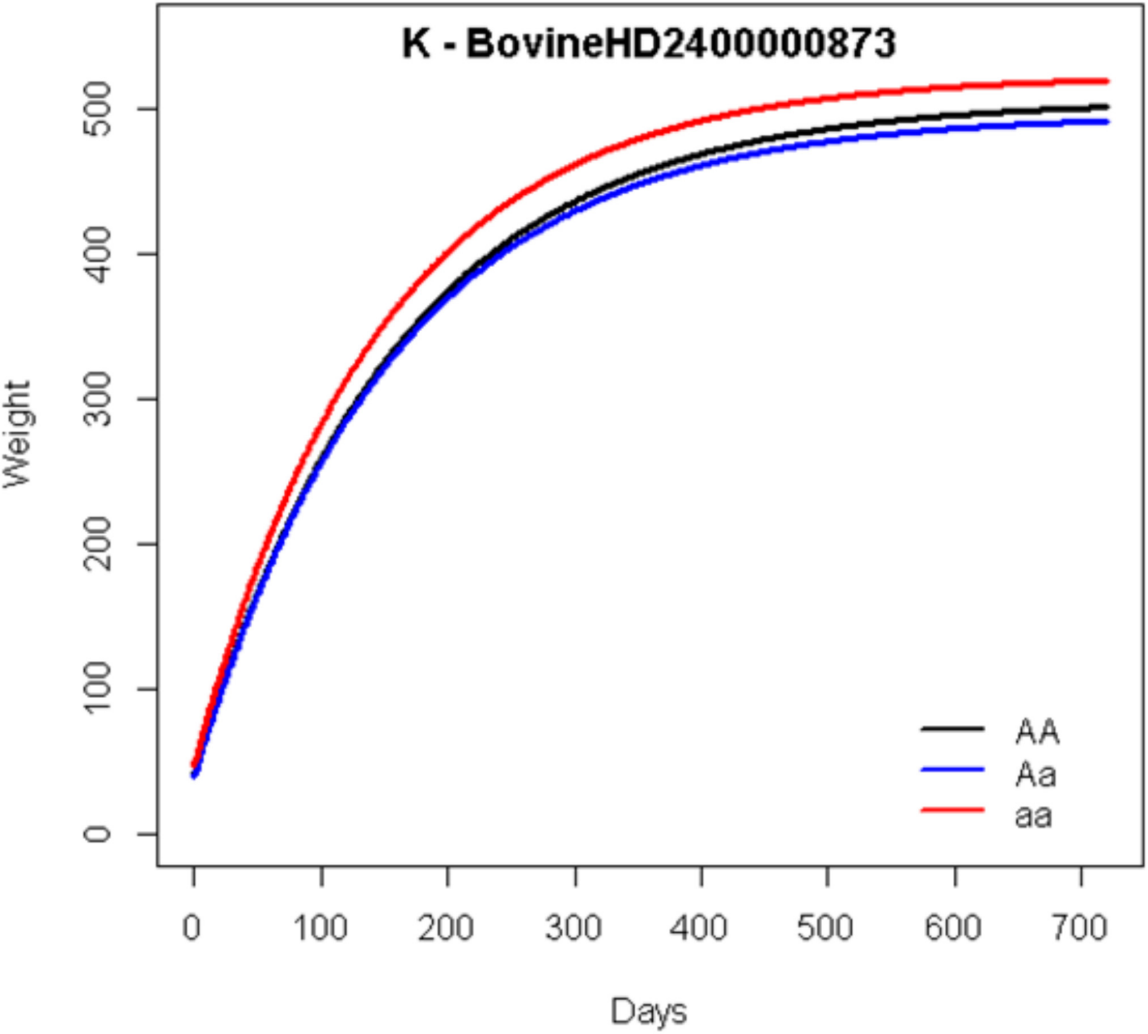
Estimated growth curves based on the Brody nonlinear model for genotypes of the most significant SNPs for maturity rate (BovineHD2000000873).

Fig 3 shows the growth curves for the genotypes of the most significant markers for mature weight (Fig 3, BovineHD0600027188) and Fig 4 for maturity rate (Fig 4, BovineHD2000000873).

For marker BovineHD0600027188the estimates of A, b, and K for the homozygous genotype were 504.59,0.92, and0.0063,respectively; for the heterozygous genotype of this marker, the estimates were 515.45,0.92, and 0.0064. Based on these estimates, the heterozygous genotype showed a higher weight at the end of the growth curve (Fig 3). According to the methodology proposed by Wu and Lin, this marker can be considered a late QTL because it remains silent during early stages and is expressed only after a particular age[7]. The two genotypes showed similar growth at early stages, but tended to diverge at later stages.

In relation to the marker BovineHD2000000873, the A, b, and K estimates for the dominant homozygote were 506.34, 0.92, and 0.0063, for the heterozygote they were 496.32, 0.92, and 0.0064,and for the recessive homozygote they were523.39, 0.91, and 0.0068. In this case, the dominant homozygote and heterozygote presented with similar behaviors in their growth curves, while the recessive heterozygote showed a higher performance (Fig 4). Additionally, although this marker might also be considered a late QTL following the [7]definitions, it is possible to note that this QTL effect started to increase earlier in comparison with the previously mentioned marker.

A haplotype block analysis was performed to uncover the genes underlying regions with large numbers of significant markers and to understand the percentage of variation explained by each significant genome region. S3 Table presents the details of the haplotype blocks, including the start and end of each region, the number of SNPs contained, and the nearest annotated genes. The number of blocks identified for the mature weight (A) and maturity rate (K) were five and nineteen, respectively. The most relevant block (number three at BTA19) for trait A explained 2.37% of the total genetic variance, while for trait K, the most important block (number 4 at BTA4) explained 2.49%. The sum of the percentage of variance explained by the five blocks for A and nineteen blocks for K were 6.5 and 18.5%, respectively.

The annotated genes that were closer to the identified relevant markers for the growth curve parameters are shown in the S1 Table and S2 Table. Thus, these genes will be discussed afterwards, in terms of their function.

In order to quantify the effectiveness of the multi-trait GWAS mixed model used in the present study, a simulation study was performed by generating replicates from permutation using the simulated data set provided by Coster et al[32]. The percentage of detected true QTLs for growth curve parameters ϕ_1_, ϕ_2_ and ϕ_3_ are presented in S3 Fig.

## Discussion

### Growth curve fitting and estimated genetic parameters

The two growth curve parameters with the most important interpretation for beef cattle breeders are the mature weight (A) and maturity rate (K); those estimates are directly obtained from fitting the nonlinear models. The Brody model was chosen as the best (Table 3) to describe the growth curve for the Brahman cattle population considered in the present study.

Although there are few studies about full growth curve analysis in Brahman cattle, different authors reported the best fit of the Brody model to analyze weight-age of this breed[37,38] and their crosses[39]. According to Forni et al. [5], in general, the traditional models (Brody, Gompertz, von Bertalanffy, and Logistic) are adequate to establish mean growth patterns and to predict the adult body weight, but the Brody model is simpler and more accurate in predicting the birth weight of animals, and therefore, it has often been used to study growth curves in beef cattle. DeNise and Brinks [40] compared the Brody and Richards growth curve models fitted to body weight of several beef cattle inbred lines, and concluded that was easier to interpret the results from the Brody equation, despite the lower number of parameters in comparison to the Richards model. Brody equation was also less sensitive to fluctuations in mature weight estimations. Furthermore, the Brody model can be applied to the estimation of A and K, even in herds where a portion of the animals are culled before adult age. Thus, these authors suggested that parameter estimates of the Brody model could be considered as traits in the selection index (by means of genetic parameter estimates), along with its corresponding economic weight, to improve the overall efficiency of beef cattle production.

The parameter estimates obtained in the present study (Table 3) were, in general, similar to the estimates from other growth curve studies in Brahman cattle. Among them, Brown et al. [39] found the average values of 556.9, 0.87, and 0.03, respectively for A, b, and K. Takahashi[37] reported that the reference mature weight (A) of Brahman cattle that should be used in feedlot diet management was around six hundred kilograms. In a crossbreeding experiment, Brown et al. [39]reported slightly lower values of A and higher values for K and b in Hereford–Brahman crosses (1/4-1/2 Brahman cattle).

In relation to the heritability estimates (0.23, 0.41, and 0.31, respectively for A, b, and K) for the growth curve parameters that were observed in the present Brahman cattle population (all considered to be moderate), we can infer that changes in the shape of these curves might be accessed by including these traits in genetic selection programs. In fact, the objective would be to obtain animals with a fast early growth rate without a dramatic increase in the adult body weight; that can be achieved by exploiting the negative nature of the genetic correlation between A and K, which was -0.84 in the present study. This negative correlation has been reported in other studies of growth curves applied to animal breeding, including the classics [1]and[40], which indicated that animals with lighter mature weights reached that weight at younger ages. Thus, in a genetic context, it is expected that animals that mature early are less likely to attain as high mature weights comparing with animals that mature slowly in early life; they are less desirable because animals with greater mature weights require more energy for maintenance and reach puberty later in life. Given the genetic correlation between parameter estimates, we believe that the multi-trait GWAS model is more feasible when it comes to identify markers that affect the growth curve of Brahman cattle.

### Genome wide association studies and gene function annotation

In summary, a joint genomic association analysis of multiple potentially correlated traits, like growth curve parameters, could be advantageous. The approach has increased the power of QTL detection as reported by Galesloot et al[41],when comparing several multivariate and univariate GWAS methods. Furthermore, these authors suggested that the multivariate methods might be able to identify genetic variants that are currently not identifiable by standard univariate analysis. For this reason, recent relevant applied GWAS in beef cattle populations[12,14]have considered this multi-trait analysis.

Quantile-quantile (Q-Q) plots were built (S1 Fig and S2 Fig) to assess the magnitude of observed associations between markers and phenotypes (growth curve parameter estimates A and K), as well as to identify potential population structure issues. Deviations from the identity line suggest that the sample contains values arising at the extremities, possibly due to a true association. Furthermore, there was no remarkable inflation in observed statistics due to relatedness and differences in the population structure, especially because the GWAS mixed model that was utilized (which includes the polygenic effect) has main advantages such as the effectiveness to control this kind of inflation.

The results of simulation study (S3 Fig) demonstrated the effectiveness of the multi-trait GWAS mixed model to detect true QTLs for growth curve parameters (ϕ_1_, ϕ_2_ and ϕ_3_) estimates. As expected, the performance to detect the main QTL (one by trait) was higher (80, 80 and 60% for ϕ_1_, ϕ_2_ and ϕ_3_) than other QTLs (50 and 48% for ϕ_1_, ϕ_2_ and ϕ_3_ in average, respectively). We would like to clarify that the original simulation study by Coster et al.[32] consists in only one data set which was replicated by permutation. Thus, the disturbances inserted to perform permutations characterize a more challenging scenario than original study. Even so, the method used in the present study outperformed the traditional Bayes B applied to the original simulated data set by Pong-Wong et al.[42]

#### Mature weight (parameter A)

The peak of significant SNPs that was observed for mature weight (A) at BTA6 was also reported in another study using beef cattle. Saatchi et al.[43]performed GWAS using 50K genotypes scored in 18,274 animals from ten north American beef cattle breeds and reported at Animal QTLdb[36]that SNPs on BTA6explained more than 10% of the additive genetic variance in mature weights of Hereford, Red Angus, and Simmental breeds. They identified 3-lead SNPs at this QTL(rs81131471 at 38.91 Mb, rs110834363 at 38.94 Mb, and rs81151923 at 39.26 Mb).Many other studies have pointed to the presence of QTL on BTA6 for body weights and growth traits[44,45]in cattle. In addition, Lu et al.[46] identified a cluster of 18 SNPs on chromosome 6 (36 Mbp—40 Mbp) that is significant for carcass weights.

The polymorphism with a higher effect (Fig 3 and S1 Table) located on BTA6 (BovineHD0600027188), associated in this study with mature weight (A), is close to the *RAB28*gene. Jiang et al.[47]indicated that *RAB*28 positively influences endothelial cell proliferation and vascular smooth muscle cells. However, these authors reported that the function of *RAB28* in mammalian cells and its role in muscle development, although proven, is still unknown and needs to be further investigated.

For the S1 Table, the *BTG1* gene (B-cell translocation gene 1) was marked by SNP BovineHD0500006477, located at BTA5:22. This gene is a member of an anti-proliferative gene family that regulates cell growth and differentiation, and it appears that *BTG1* acts as a myogenic inductor[48,49] in a broad study about muscle differentiation, thus showing that *BTG1* is an important coactivator involved in the regulation of myoblast differentiation. In addition, *BTG1* not only stimulates the activity of myogenic factors, but also the activity of nuclear receptors already known to be positive myogenic regulators.

The *IL2* gene (Interleukin–2) was related to the BovineHD0600027154, BovineHD0600027161, BovineHD0600027170, and BovineHD0600027188 markers that are also represented in the peak of BTA6 (Fig 1). Although, several studies in different beef cattle populations reported that the region of this gene on chromosome 6 harbors quantitative trait loci (QTL) affecting fetal growth[44,50]. In general, the protein (cytokine) encoded by this gene is required for T-cell proliferation and other activities crucial to regulation of the immune response[51].This gene has also been associated with tick-resistance in Brahman cattle [52].

The significant BovineHD3000046685 marker on chromosome X (Fig 1) for mature weight was identified close to the*APEX2* gene. Ide et al.[53] compared *APEX2-*null mice with the wild type, and observed that *APEX2*-null mice body weights were about 80%of the wild-type male littermates at birth; this tendency persisted into childhood and adulthood, thereby indicating that all developing embryos, infants, and adults of *APEX2*-null mice may somehow be retarded in terms of growth.

#### Maturity rate (parameter K)

Related to maturity rate (K), the peak identified in the Manhattan plot at chromosome 20 had 64 significant SNPs, 8 of them building 3 blocks, which explained 3% of the additive genetic variance of the characteristic (S3 Table). The other 56 were not in linkage disequilibrium. All significant polymorphisms identified on chromosome 20 accounted for 26% of the total additive genetic variance of the characteristic (S3 Table).

The *TMEM18* gene (Transmembrane Protein 18) was marked by a SNP at BTA1 associated with trait K (maturity rate) and has been reported to be associated with growth traits and obesity[54,55]. The association analysis of genotypes in the single and combined SNPs located in the exonic region of the *TMEM18* gene revealed a consistent effect on growth traits in Nanyang cattle, especially on body weight, body height, hucklebone width, and average daily gain in cattle aged 6 months[56]. As already known, mutations in or near the *TMEM18* gene were associated with larger waist circumferences and total body fat in humans [57]. There are studies that also revealed that novel SNPs near the *TMEM18* gene had a significant association with body weight in rats [55].

One of the genes that was found on BTA20:8Mb was *SMN1*, which is involved with skeletal muscle development in murines [58]. The effect of *SMN* gene mutations in the degeneration of muscle fibers is supported by results obtained in mice with a deletion of *SMN* exon 7 restricted to skeletal muscle[59]. Another gene acting on BTA20:8Mb is *MSX2*, which is related to bone growth and ectodermal organ formation in mice [60].

The *SFRP2* gene is a protein-coding gene that was marked close to significant markers on BTA17 for both traits (mature weight and maturity rate) in Fig 1 and Fig 2. This gene has been reported to be related to embryonic organogenesis in mammals [61], and consists of relevant information, since polymorphisms that affect multiple traits confirm the complexity of growth processes in beef cattle [14].

### Future Implications

The GWAS results do not provide direct functional information regarding the most relevant identified loci (individual significant SNP markers). Thus, additional analysis, like the SNP-derived gene function annotation that was used in the present study, is needed to identify the candidate genes and their role in the post-natal growth trajectory of Brahman cattle. However, a complementary study associated with the validation of GWAS candidate genes, like expression analyses, re-sequencing of genes, and haplotype blocks, may be considered in the future. Initially, we may propose a quantitative real-time PCR (qPCR) under contrasting defined environmental conditions. In the context of this study, these conditions can be supported by groups of animals that are genetically different in relation to the growth curve shapes. These groups can be selected by means of the predicted breeding values for the parameter estimates (A, b, and K) generated by the utilized multi-trait mixed model methodology.

Other methods to validate or refine the identified candidate genes in future studies can be done directly by a re-sequencing approach. The advent of next-generation sequencing (NGS), and the highly decreased whole-genome sequencing associated costs, allow us to sequence specific genome regions (related to genes of interest) of a few contrasting individuals and to access, for example, by alignment algorithms, causal polymorphisms underlying these regions[62,63].

## Conclusion

Of the five most used nonlinear growth models (Brody, Gompertz, von Bertalanffy, Logistic, and Richards), the Brody model was the most appropriate model to summarize growth curve behaviors and describe the growth curve of Brahman cattle considered in the present study. The heritability values for the parameter estimates of mature weight (A) and maturity rate (K) indicated that these traits can be a feasible alternative for breeding programs aiming to change the shape of growth curves within genetic improvement programs. The use of estimated parameters with biological interpretations extracted from the best model and treated as phenotypes in a multi-trait GWAS was efficient at indicating SNP-derived candidate genes with functions related to biological processes of growth in beef cattle. New candidate regions for growth traits were detected, and some of them have interesting biological functions. Future studies targeting these areas could provide further knowledge to uncover the genetic architecture underlying growth traits in Brahman cattle.

## Supporting Information

S1 Datafile File containing identification of all animals, pedigree information, breed information, year/month/day of birth, farm code where they born and farm code where they were relocated after post weaning, and weight measures at different ages. (CSV)

S1 Fig QQ plot for mature weight (A) based on t-student test. (TIF)

S2 Fig QQ plot for maturity rate (K) based on t-student test. (TIF)

S3 Fig Performance of the multi-trait GWAS mixed model to detect QTL for the ϕ_1_by using simulated data for parametersϕ_1_, ϕ_2_ and ϕ_3_
(TIF)

S1 Script Script built in order to test nonlinear models. (TXT)

S1 Table Significant SNPs for mature weight (A) sorted by chromosome and then pvalue. (PDF)

S2 Table Significant SNPs for maturity rate (K) sorted by chromosome and then pvalue. (PDF)

S3 Table Blocks formed by linkage disequilibrium among the 167 significant SNPs for A (mature weight) and 262 significant SNPs for K (maturity rate). (PDF)

S4 Table Comparison between four nonlinear growth models (Gompertz, Logistic, Brody, and von Bertalanffy) using different covariance matrix structures (diagonal, corAR1, corCAR1, corARMA1). Estimates for growth curve parameters: mature weight (A), scale (b), and maturity rate (K). Goodness of fit measures: LOGLIK, Akaike Information Criterion (AIC), and Bayesian Information Criterion (BIC), using *nlme* function by R software.
(PDF)
