# Supplementary figures and images for: Multi-Trait GWAS and New Candidate Genes Annotation for Growth Curve Parameters in Brahman Cattle
Source: PLoS One. 2015 Oct 7;10(10):e0139906. doi: 10.1371/journal.pone.0139906 (PMC4622042; doi:10.1371/journal.pone.0139906)

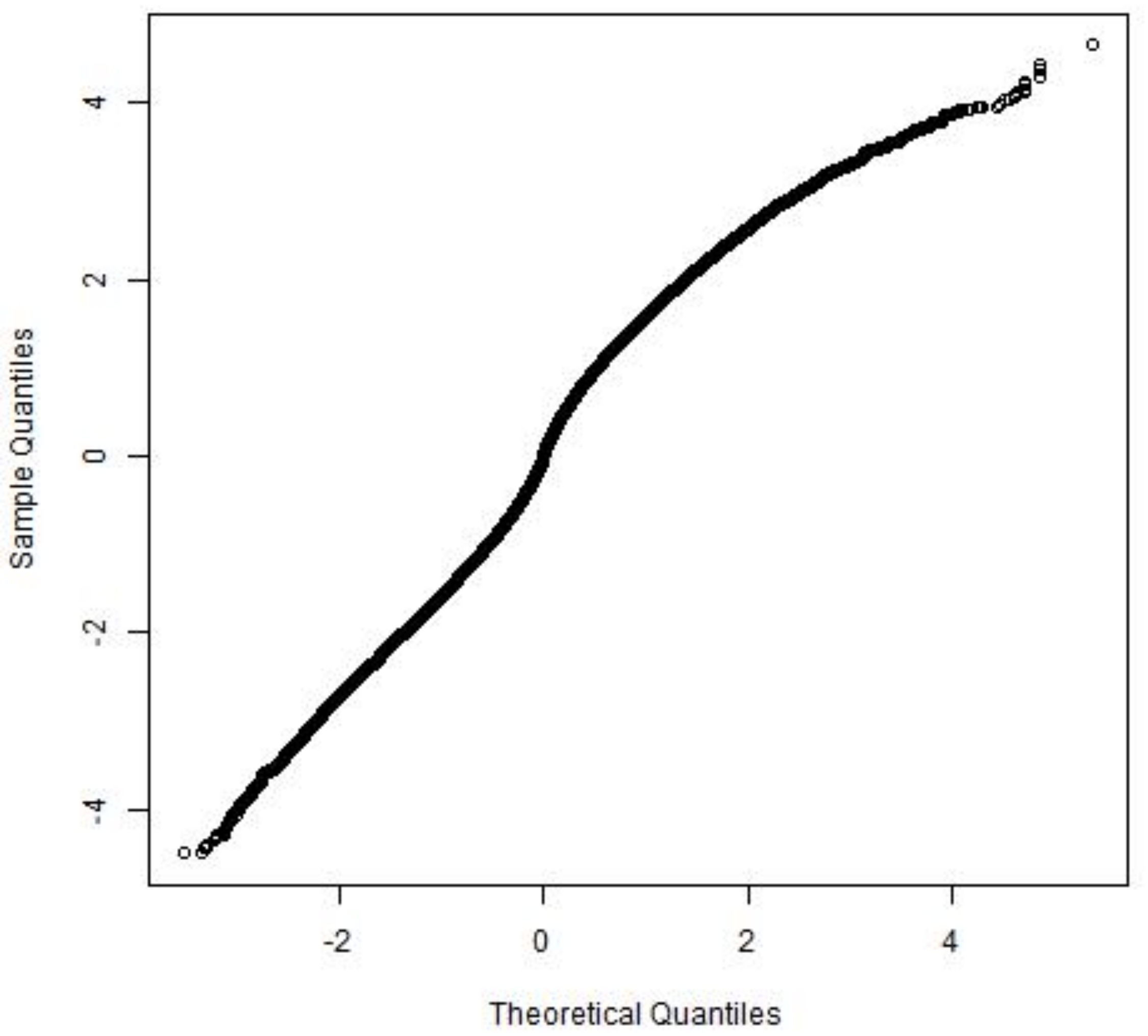

Supplement: S1 Fig — (TIF) [file pone.0139906.s002.tif]

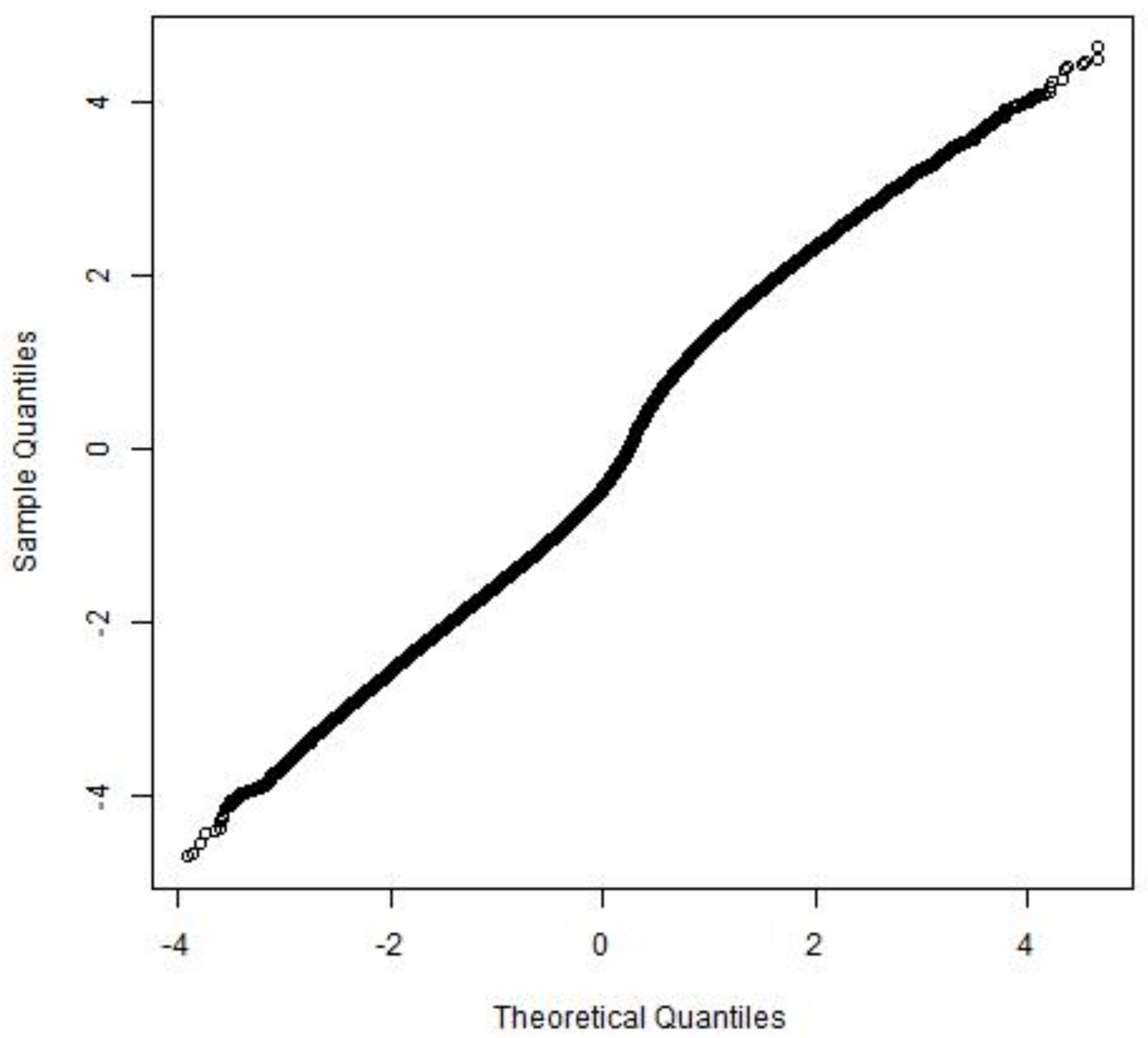

Supplement: S2 Fig — (TIF) [file pone.0139906.s003.tif]

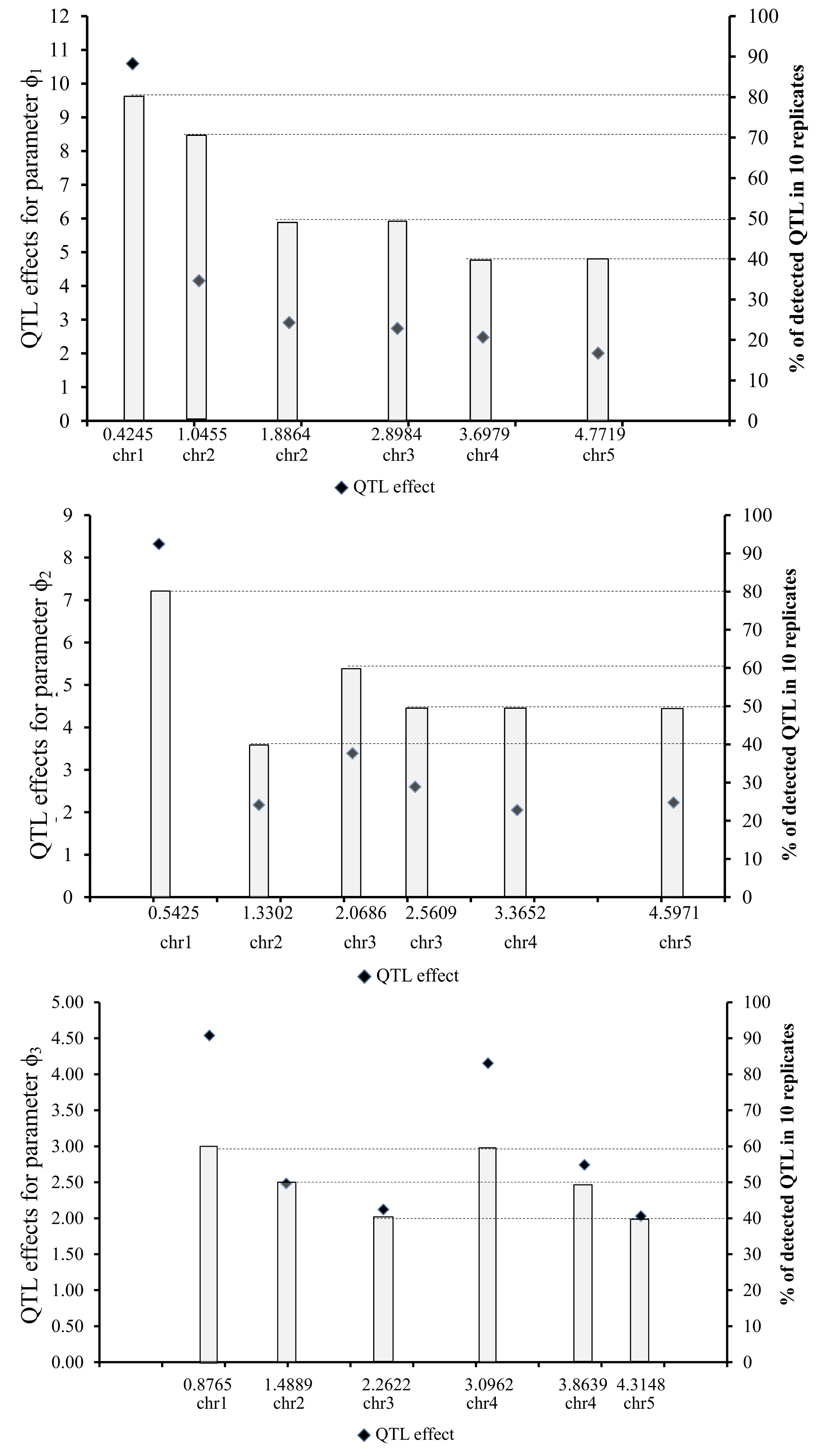

Supplement: S3 Fig — (TIF) [file pone.0139906.s004.tif]
