# Supplementary material for: Multi-Trait GWAS and New Candidate Genes Annotation for Growth Curve Parameters in Brahman Cattle
Source: PLoS One. 2015 Oct 7;10(10):e0139906. doi: 10.1371/journal.pone.0139906 (PMC4622042; doi:10.1371/journal.pone.0139906)
Supplement: S1 Table — (PDF) [file pone.0139906.s006.pdf]

S1 Table. Table listing markers with no strong linkage disequilibrium (LD) with other markers and its nearest genes for each chromosome associated to mature weight (A). Markers were sorted by chromosome and then *P*-value and so the table summarizes the most significant SNPs with no strong LD for each chromosome

Markers	Chr	pvala	Position	Chr_start	Chr_stop	Feature_name	Distance	%Var
BovineHD0200032095	2	0.00015114	111355325	111309377	111347961	LOC618198	7364	0.47500087
BovineHD0200033231	2	0.00021635	115139245	115124265	115156084	LOC518699	0	0.43087413
BovineHD0200032094	2	0.00021802	111354445	111309377	111347961	LOC618198	6484	0.44546294
BovineHD0200032097	2	0.00021802	111358765	111309377	111347961	LOC618198	10804	0.44546294
ARS-BFGL-NGS-118904	2	0.00035589	53951533	54147528	54248399	LOC782540	195995	0.46604181
Hapmap35744-SCAFFOLD60587_8279	2	0.00075341	111361143	111309377	111347961	LOC618198	13182	0.37263542
BovineHD0200015359	2	0.00089504	53915714	54147528	54248399	LOC782540	231814	0.41055928
BovineHD0300016037	3	6.66E-05	53158505	53205967	53257297	SH3GLB1	47462	1.04138335
BovineHD0300009685	3	0.00082783	30778559	30800991	30802100	LOC539739	22432	0.38843028
BovineHD0400007804	4	5.13E-05	26988234	26698950	26708119	LOC783441	280115	0.49949804
BovineHD0400007849	4	5.13E-05	27135030	27280043	27280537	LOC538985	145013	0.49949804
BovineHD0400008013	4	5.13E-05	27568744	27448443	28024227	LOC535415	0	1.82376906
BovineHD0400009420	4	5.13E-05	33197944	33225289	33366637	LOC781830	27345	1.98851301
BTB-01114756	4	5.13E-05	27560851	27448443	28024227	LOC535415	0	0.6656463
BovineHD0400034063	4	9.75E-05	117064985	110843368	110870369	LOC520408	6194616	0.43391181
BovineHD4100002757	4	0.00014143	30202937	30365441	30433571	LOC523019	162504	0.45978267
BovineHD0400034397	4	0.00016103	117668183	110843368	110870369	LOC520408	6797814	0.32829866
BovineHD0400034928	4	0.00025699	119234224	110843368	110870369	LOC520408	8363855	0.46917421
Hapmap54229-rs29017613	4	0.00036594	30200987	30365441	30433571	LOC523019	164454	0.46186836
BovineHD0400034134	4	0.00044052	117215749	110843368	110870369	LOC520408	6345380	0.46143981
BovineHD0400034145	4	0.00044052	117240334	110843368	110870369	LOC520408	6369965	0.46143981
BovineHD0400008147	4	0.0008611	28288554	28331833	28510179	LOC618384	43279	0.34810785
BovineHD0500006477	5	4.52E-06	22373212	22559940	22562652	BTG1	186728	1.28172538
BovineHD0500006481	5	4.52E-06	22388982	22559940	22562652	BTG1	170958	1.28172538
BovineHD0500032881	5	0.00050233	113878281	113885881	113894615	LOC789821	7600	1.33813115
BovineHD0500023351	5	0.00052417	82607562	82292908	82310569	MGC137384	296993	0.45087779

BovineHD0500023371	5	0.00052417	82640169	82292908	82310569	MGC137384	329600	0.45087779
BovineHD0500023380	5	0.00052417	82652835	82292908	82310569	MGC137384	342266	0.45087779
BovineHD0500023383	5	0.00052417	82662475	82292908	82310569	MGC137384	351906	0.45087779
BovineHD0500023373	5	0.00072325	82643997	82292908	82310569	MGC137384	333428	0.45717603
BovineHD0500023411	5	0.00072932	82754564	82292908	82310569	MGC137384	443995	0.43796097
BovineHD0500005741	5	0.00077129	19806551	19763156	19788394	KITLG	18157	1.138398
BovineHD0500006162	5	0.00077129	21362355	21216198	21324755	LOC782975	37600	1.138398
BovineHD0500006167	5	0.00077129	21367655	21216198	21324755	LOC782975	42900	1.138398
BovineHD0500006182	5	0.00077129	21415740	21216198	21324755	LOC782975	90985	1.138398
BovineHD0500023546	5	0.00080975	83165869	83580272	83581359	LOC541057	414403	0.46851666
BovineHD0500023376	5	0.00091239	82648706	82292908	82310569	MGC137384	338137	0.4462513
BovineHD0600027188	6	9.79E-08	97907606	97851185	97854333	IL2	53273	1.53578042
BovineHD0600027517	6	1.32E-06	98915874	98809532	98824808	MGC127281	91066	0.75954816
BovineHD0600027518	6	1.32E-06	98921247	98809532	98824808	MGC127281	96439	0.75954816
BovineHD0600027520	6	1.32E-06	98924679	98809532	98824808	MGC127281	99871	0.75954816
BTB-01204750	6	2.91E-06	13417489	13210891	13211865	LOC785498	205624	1.31825597
Hapmap32223-BTC-044639	6	4.20E-06	102215038	102478990	102566753	LOC614799	263952	0.77785153
BovineHD0600027170	6	5.53E-06	97859333	97851185	97854333	IL2	5000	0.55720457
BovineHD0600027507	6	9.74E-06	98896708	98809532	98824808	MGC127281	71900	0.6182961
BovineHD0600027154	6	1.97E-05	97789599	97851185	97854333	IL2	61586	0.50895535
BovineHD0600028652	6	2.37E-05	102886663	102911068	103023999	LOC517240	24405	0.72556314
BovineHD0600028665	6	2.37E-05	102944964	102911068	103023999	LOC517240	0	0.71143623
ARS-BFGL-NGS-26389	6	3.16E-05	101096589	101230402	101230554	LOC785939	133813	0.77172786
BovineHD0600028633	6	4.42E-05	102840357	102738182	102865778	MGC142642	0	0.47414487
BovineHD0600028676	6	5.32E-05	102991379	102911068	103023999	LOC517240	0	0.6364964
BTA-121434-no-rs	6	6.22E-05	100428424	100742310	100755654	LOC785837	313886	0.57788249
BovineHD0600032353	6	7.56E-05	114176857	110935645	111033198	MGC128932	3143659	0.57143111
BovineHD0600032342	6	8.23E-05	114156474	110935645	111033198	MGC128932	3123276	0.59638995
BovineHD0600030324	6	8.92E-05	107618872	107605618	107619569	LOC504957	0	0.70134148
BovineHD0600032427	6	9.76E-05	114436483	110935645	111033198	MGC128932	3403285	0.67811755
BovineHD4100005507	6	0.00011905	102790401	102738182	102865778	MGC142642	0	0.4356367

BovineHD0600032313	6	0.00016332	114098001	110935645	111033198	MGC128932	3064803	0.5894787
BovineHD0600011495	6	0.00017784	42277712	42172542	42274983	MGC140504	2729	0.43314805
ARS-BFGL-NGS-71333	6	0.00017968	89075993	88374935	88921637	ARHGAP24	154356	0.41884506
BovineHD0600031012	6	0.0001878	110151219	110147359	110172120	LOC782761	0	0.60386413
BovineHD0600011787	6	0.00020596	43433730	43379453	43490213	MGC128782	0	0.68426294
BovineHD0600025472	6	0.0002282	92705860	92427336	92458278	SLC10A6	247582	0.27196106
BovineHD0600025838	6	0.0002282	93393456	93379108	93418563	LOC539372	0	0.27196106
BovineHD0600028183	6	0.00025662	100929172	100757430	100793611	MGC142451	135561	0.58635883
BovineHD0600028705	6	0.00029303	103102344	103033235	103089693	LOC519695	12651	0.42737757
BovineHD0600011756	6	0.00030444	43368544	43379453	43490213	MGC128782	10909	0.71797462
BovineHD0600011759	6	0.00030444	43374796	43379453	43490213	MGC128782	4657	0.71797462
BovineHD0600011775	6	0.00030444	43402739	43379453	43490213	MGC128782	0	0.71797462
BovineHD4100005509	6	0.00036122	102793498	102738182	102865778	MGC142642	0	0.39500865
BovineHD0600032432	6	0.00039493	114454346	110935645	111033198	MGC128932	3421148	0.43595933
BovineHD0600027161	6	0.00039846	97813656	97851185	97854333	IL2	37529	0.4814652
BovineHD0600026979	6	0.00040361	97178687	97145283	97214148	LOC783930	0	0.5715323
BovineHD0600027030	6	0.00040835	97314118	97258057	97299086	MGC127353	15032	0.35908327
BovineHD0600025830	6	0.0004532	93368113	93354518	93371616	CCDC98	0	0.24723032
BovineHD0600032318	6	0.00052018	114116979	110935645	111033198	MGC128932	3083781	0.48370953
BovineHD0600032358	6	0.00058042	114193732	110935645	111033198	MGC128932	3160534	0.51502728
BovineHD0600019898	6	0.0009009	71449954	71244398	71818706	LEC3	0	0.38027543
BovineHD0600030287	6	0.00091154	107538017	107516063	107541102	LOC505477	0	0.53282646
BovineHD0600030313	6	0.00091154	107607570	107605618	107619569	LOC504957	0	0.53282646
BovineHD0600027033	6	0.0009781	97316958	97258057	97299086	MGC127353	17872	0.34515742
BovineHD0800020555	8	2.24E-05	68473880	68417099	68530970	LOC532684	0	0.8454201
BovineHD0800024309	8	0.00015462	81841082	81741733	81790423	LOC781205	50659	0.29115993
BTB-00362255	8	0.00015462	81866074	81741733	81790423	LOC781205	75651	0.29115993
BovineHD0800024222	8	0.00036377	81537751	81413255	81449593	LOC538667	88158	0.49912374
BovineHD0800004640	8	0.00043759	14831058	14720502	14829375	LOC785941	1683	0.65822781
BovineHD0800004667	8	0.00043759	14919451	14997823	15003175	LOC786143	78372	0.65822781
BovineHD0800004669	8	0.00043759	14925557	14997823	15003175	LOC786143	72266	0.65822781

BovineHD0800004670	8	0.00043759	14927629	14997823	15003175	LOC786143	70194	0.65822781
BovineHD0800004694	8	0.00043759	15022993	15034413	15105042	MGC133601	11420	0.65822781
BovineHD0800018125	8	0.00050776	60719561	60665163	60668611	LOC786270	50950	0.56204591
BovineHD0800018155	8	0.00050776	60789911	60665163	60668611	LOC786270	121300	0.56204591
BovineHD0800005595	8	0.00059102	17873442	18379284	18639897	LOC786878	505842	1.19077782
BovineHD0800005603	8	0.00059102	17898087	18379284	18639897	LOC786878	481197	1.07226708
BovineHD0800016657	8	0.00065653	55223436	55152915	55245002	LOC512888	0	0.55217624
BovineHD0800003847	8	0.00077129	11755496	11418713	11422744	LOC783420	332752	0.91119899
BovineHD0800020836	8	0.00077129	69336118	69363553	69400275	WDR40A	27435	0.45608008
BovineHD0900021870	9	0.00014693	78450681	78466302	78648155	LOC535975	15621	0.83783394
BovineHD0900021873	9	0.00014693	78460717	78466302	78648155	LOC535975	5585	0.83783394
BovineHD1000000261	10	0.00030061	890364	792166	803266	LOC785663	87098	0.78666221
BovineHD1100001456	11	9.77E-05	3990896	3960714	4017307	LOC536340	0	0.61707861
BovineHD1100004656	11	0.00044567	14260840	14257232	14269244	LOC511001	0	0.43659509
BovineHD1100009735	11	0.00045305	32353685	32398354	32398725	LOC781388	44669	0.38654677
BovineHD1100001449	11	0.0007288	3962378	3960714	4017307	LOC536340	0	0.34484303
BovineHD1100014200	11	0.0007957	48397856	48344048	48636034	LOC538058	0	0.36247394
BovineHD1100014202	11	0.0007957	48399883	48344048	48636034	LOC538058	0	0.36247394
BovineHD1100022797	11	0.00091135	79514927	79212034	79212441	LOC788469	302486	0.38659959
BovineHD1200011359	12	0.00018187	39953697	40432286	40432651	LOC789001	478589	0.56395312
BovineHD1200011989	12	0.00047293	42760473	42701798	42932277	LOC524913	0	0.37993616
BovineHD1200001981	12	0.00071777	6914353	7191123	7193153	LOC786945	276770	0.23144934
BovineHD1200002015	12	0.00071777	7086000	7191123	7193153	LOC786945	105123	0.46216664
BovineHD1300004807	13	0.0009638	16852876	16826246	16849176	LOC512694	3700	0.83076982
BTA-05407-rs29019567	15	0.00054602	20610552	20674135	20684338	LOC615090	63583	0.42255197
BovineHD1600021941	16	0.00082762	76187883	72823891	72834138	LOC509797	3353745	0.50188827
BovineHD1600021942	16	0.00082762	76195207	72823891	72834138	LOC509797	3361069	0.50188827
BovineHD1600021944	16	0.00082762	76200952	72823891	72834138	LOC509797	3366814	0.50188827
BovineHD1700013129	17	0.00018946	46928941	46685030	46934790	LOC540363	0	0.59646967
BovineHD1700000867	17	0.00098419	3656789	3641911	3650486	SFRP2	6303	0.46014539
ARS-BFGL-NGS-19022	18	6.73E-06	65551047	62889251	62891477	LOC790330	2659570	1.09716435

BovineHD1800013712	18	0.00027609	46597873	46568328	46582017	LOC521171	15856	0.53817642
BovineHD1800009739	18	0.0005343	32239978	32230082	32269399	ATP6D	0	0.49691078
BovineHD1800018806	18	0.00097231	64768873	62889251	62891477	LOC790330	1877396	0.29958816
BovineHD1900017112	19	9.10E-06	59917525	60076935	60086036	LOC787823	159410	0.39572451
BovineHD1900001049	19	0.0001571	4407360	3992700	4143802	LOC781740	263558	0.81062732
BovineHD4100014346	19	0.00029976	57020892	56938203	57025786	LOC789737	0	0.43813463
BovineHD1900017042	19	0.00035442	59664174	60076935	60086036	LOC787823	412761	0.51197017
BovineHD1900017051	19	0.00035442	59705455	60076935	60086036	LOC787823	371480	0.51197017
BovineHD1900017056	19	0.00035442	59740822	60076935	60086036	LOC787823	336113	0.51197017
BovineHD1900017058	19	0.00035442	59745154	60076935	60086036	LOC787823	331781	0.51197017
BovineHD1900017026	19	0.00055158	59614413	59161156	59163432	LOC790129	450981	0.32002279
BovineHD1900018660	19	0.00067282	9006976	8974082	9194966	LOC537257	0	0.44396043
BovineHD2000001917	20	2.91E-06	6034518	6022661	6023433	LOC783392	11085	1.31825597
BovineHD2000009323	20	0.00043754	32481431	32421715	32542203	LOC535804	0	0.69385379
BovineHD2100001787	21	2.91E-06	8181149	8178786	8320248	LOC407231	0	0.87930059
BovineHD2100000447	21	8.95E-05	3016617	2907014	3132629	LOC510723	0	0.68517099
BovineHD2200017289	22	8.76E-05	59571549	59524064	59527996	LOC617772	43553	0.88089923
BovineHD2200000864	22	0.00010653	3194730	3414073	3505940	LOC535376	219343	0.61770325
BovineHD2200013732	22	0.00018848	47701782	47635366	47708278	LOC785746	0	0.75084072
BovineHD2200016462	22	0.00049046	57103659	57109237	57171804	LOC519899	5578	0.48764421
BovineHD2200012819	22	0.00079838	44370842	43492256	44563593	LOC537173	0	0.79571041
BovineHD2200013582	22	0.00079838	47271241	47273183	47295840	LOC783987	1942	0.68239779
BTB-00848517	22	0.00079838	44430993	43492256	44563593	LOC537173	0	0.79571041
BovineHD2300009168	23	2.15E-05	31762663	31688831	31691301	SOX4	71362	0.99524106
BovineHD2300009172	23	2.15E-05	31778851	31688831	31691301	SOX4	87550	0.99524106
BovineHD2300001341	23	3.50E-05	5584389	5474689	5585785	LOC618855	0	0.64884986
BovineHD2300009167	23	0.00014561	31747290	31688831	31691301	SOX4	55989	0.8098724
BovineHD2300001187	23	0.00041268	5025076	5022572	5028461	BOLA-DYA	0	0.5204639
BovineHD2300011460	23	0.00045433	39654679	39628234	39920734	MGC151783	0	0.45199863
BovineHD2300001338	23	0.00056928	5575255	5474689	5585785	LOC618855	0	0.36511824
BovineHD2300005631	23	0.00093845	21402035	21369764	21399894	LOC786695	2141	0.50038524

BovineHD2400010368	24	2.91E-06	37816668	37863264	37864256	LOC786975	46596	0.43988193
BovineHD2700000762	27	2.91E-06	2279279	2307555	2308325	LOC517971	28276	0.43988193
BovineHD2700000770	27	2.91E-06	2306884	2307555	2308325	LOC517971	671	0.43988193
BovineHD2700000774	27	2.91E-06	2331788	2307555	2308325	LOC517971	23463	0.43988193
BovineHD2700000775	27	2.91E-06	2339835	2307555	2308325	LOC517971	31510	0.43988193
BovineHD2700011135	27	0.00029629	38582346	38304464	38352009	LOC787377	230337	0.35406176
BovineHD2700011124	27	0.00029815	38534442	38304464	38352009	LOC787377	182433	0.33465188
BovineHD2700012060	27	0.00037578	41551568	41172444	41289736	LOC784356	261832	0.37012178
BovineHD2700010770	27	0.00047067	37447990	37181818	37560782	LOC531459	0	0.38247737
BovineHD2700004261	27	0.00050401	14650855	14560219	14653912	LOC790088	0	0.43243618
BovineHD2700012073	27	0.0005194	41618491	41172444	41289736	LOC784356	328755	0.43069712
BovineHD2700010776	27	0.00066828	37470446	37181818	37560782	LOC531459	0	0.36162767
BovineHD2700011611	27	0.00076589	40047751	40139624	40621183	LOC784326	91873	0.38563058
BovineHD2700011842	27	0.00076589	40780949	40139624	40621183	LOC784326	159766	0.38563058
BovineHD3000019003	30	0.00029678	65152485	65270083	65270590	LOC784159	117598	0.21897076
BovineHD3000019030	30	0.00029678	65221973	65270083	65270590	LOC784159	48110	0.21897076
BovineHD3000046685	30	0.0008286	64847319	64815965	64823638	APEX2	23681	0.18399554
