# Supplementary material for: Multi-Trait GWAS and New Candidate Genes Annotation for Growth Curve Parameters in Brahman Cattle
Source: PLoS One. 2015 Oct 7;10(10):e0139906. doi: 10.1371/journal.pone.0139906 (PMC4622042; doi:10.1371/journal.pone.0139906)
Supplement: S2 Table — (PDF) [file pone.0139906.s007.pdf]

S2 Table. Table listing markers with no strong linkage disequilibrium (LD) with other markers and its nearest genes for each chromosome associated to maturity rate (K). Markers were sorted by chromosome and then *P*-value and so the table summarizes the most significant SNPs with no strong LD for each chromosome

Markers	Chr	pvalk	Position	Chr_start	Chr_stop	Feature_name	Distance	%Var
BovineHD0100011757	1	4.29E-05	41233947	41326013	41409064	LOC781270	92066	0.52876136
BovineHD0100008623	1	0.000124803	29084731	28787675	28787935	LOC781386	296796	0.3649257
BovineHD0100044544	1	0.000164868	153050619	146046628	146050938	TMEM18	6999681	0.3266512
BovineHD0100044549	1	0.000164868	153056651	146046628	146050938	TMEM18	7005713	0.3266512
BovineHD0100008628	1	0.000190022	29097600	28787675	28787935	LOC781386	309665	0.33715577
BovineHD0100015793	1	0.000270589	56125570	55682802	55683963	LOC781645	441607	0.31311612
BovineHD0100008629	1	0.000369157	29098485	28787675	28787935	LOC781386	310550	0.31318547
BovineHD0100033663	1	0.000436224	119188091	119117039	119157083	LOC539843	31008	0.27232588
ARS-BFGL-BAC-16186	1	0.000541242	157395805	146046628	146050938	TMEM18	11344867	0.3340821
BovineHD0100046943	1	0.000822347	31496031	31490853	31495762	LOC790863	269	0.27108213
BovineHD0100009290	1	0.000859598	32059968	32138605	32221912	LOC613799	78637	0.24482289
BovineHD0200036282	2	2.50E-05	124919532	124853361	124923186	MGC139675	0	0.49780729
BovineHD0200001042	2	4.61E-05	3904111	3895689	3907731	LOC782818	0	0.40647095
BovineHD0200029813	2	7.46E-05	103686803	103535205	103811354	LOC615765	0	0.93708227
BovineHD0200037821	2	0.000455093	130161730	125773468	125794102	LOC616377	4367628	0.49378086
BovineHD0200035678	2	0.00060923	123045219	123015389	123032153	LOC789096	13066	0.43103756
BovineHD0200029833	2	0.000636795	103746725	103535205	103811354	LOC615765	0	0.45246459
BovineHD0200027147	2	0.000732272	94542687	94337227	94728448	LOC782360	0	0.24482496
BovineHD0200027159	2	0.000732272	94557690	94337227	94728448	LOC782360	0	0.24482496
BovineHD0200007282	2	0.000896404	25344654	25523332	25581013	LOC540766	178678	0.30949236
BovineHD0200001550	2	0.000918976	5354593	5156851	5176584	LOC786192	178009	0.32414999
BovineHD0300005371	3	2.76E-05	16503436	16482571	16493504	S100A10	9932	1.3095062
BovineHD0300001240	3	5.87E-05	3991918	3975382	3996648	LOC508183	0	0.48408682
BovineHD0300011554	3	0.000259163	37226889	37050029	37050432	LOC787616	176457	0.34680662
BovineHD0300011598	3	0.000852979	37411646	37495596	37496010	LOC787637	83950	0.3059741
INRA-453	3	0.000862051	19248260	19249447	19252795	LOC518825	1187	0.35534866
BovineHD0400031190	4	1.67E-05	108975638	108994462	108995968	LOC614377	18824	0.40581231

BovineHD0400031404	4	0.00038413	109727854	109593798	109672415	LOC512582	55439	0.31394745
BovineHD0400005433	4	0.000464613	18168180	18113694	18587211	LOC517284	0	0.30480253
BovineHD0400034063	4	0.000499149	117064985	110843368	110870369	LOC520408	6194616	0.25806934
BovineHD0400031871	4	0.000711273	111101754	110843368	110870369	LOC520408	231385	0.23728594
BovineHD0400028185	4	0.000752304	100615708	99955990	100382392	LOC788066	233316	0.33893054
BovineHD0400010516	4	0.000813287	37502618	37572976	37707766	LOC537777	70358	0.31920266
BovineHD0400031372	4	0.000876091	109633108	109593798	109672415	LOC512582	0	0.30999133
BovineHD0500036248	5	5.87E-05	105051999	104992297	105054740	LOC615834	0	0.3808224
BovineHD4100004090	5	9.98E-05	110251486	110119934	110358740	LOC509011	0	0.4362621
BovineHD0500032843	5	0.0001055	113726014	113716432	113731138	LOC790892	0	0.55246201
BovineHD0500032844	5	0.0001055	113730594	113716432	113731138	LOC790892	0	0.55246201
BovineHD0500029899	5	0.000107182	104496844	104415828	104438717	MGC142404	58127	0.45913916
ARS-BFGL-NGS-42687	5	0.00012728	105288314	105223293	105412829	LOC540132	0	0.41113519
BovineHD0500017023	5	0.00017578	60721425	60846730	60888845	LOC539655	125305	0.54969866
BovineHD0500031339	5	0.000209059	108793263	108766083	108812776	LOC535381	0	0.49518029
BovineHD0500031342	5	0.000209059	108803665	108766083	108812776	LOC535381	0	0.49518029
BovineHD0500029906	5	0.000252874	104548821	104415828	104438717	MGC142404	110104	0.46652934
BovineHD0500019552	5	0.00027441	69831255	69928146	69931199	MGC137188	96891	0.32477711
BovineHD0500019556	5	0.00027441	69841335	69928146	69931199	MGC137188	86811	0.32477711
BovineHD0500019572	5	0.00027441	69885003	69928146	69931199	MGC137188	43143	0.32477711
BovineHD0500019575	5	0.00027441	69894023	69928146	69931199	MGC137188	34123	0.32477711
BovineHD0500031445	5	0.000376375	109149853	109149240	109159647	JOSD1	0	0.4570919
BovineHD0500032584	5	0.000438131	112839060	112609746	112832121	LOC523518	6939	0.41761513
BovineHD0500017160	5	0.000660686	61262958	61257822	62047789	LOC516896	0	0.39315005
BovineHD0600013748	6	0.000251628	49942445	51163313	51263216	LOC781512	1220868	0.40124513
BovineHD0600001316	6	0.000291331	4994214	4877795	4878609	LOC781502	115605	0.26647554
BovineHD0600001303	6	0.000364399	4942854	4877795	4878609	LOC781502	64245	0.30796532
BovineHD0600001333	6	0.000386404	5065951	4877795	4878609	LOC781502	187342	0.25475438
BovineHD0600001335	6	0.000386404	5071197	4877795	4878609	LOC781502	192588	0.25475438
BovineHD0600001286	6	0.000417167	4876731	4877795	4878609	LOC781502	1064	0.24891272
BovineHD0600001287	6	0.000417167	4880955	4877795	4878609	LOC781502	2346	0.24891272
BovineHD0600001323	6	0.000427537	5028589	4877795	4878609	LOC781502	149980	0.24703465

BovineHD0600001307	6	0.000480994	4950064	4877795	4878609	LOC781502	71455	0.24778269
BovineHD0600001346	6	0.000483908	5118327	4877795	4878609	LOC781502	239718	0.2432862
BovineHD0600001252	6	0.00048459	4786967	4877795	4878609	LOC781502	90828	0.28479981
BovineHD0600001283	6	0.000609964	4862921	4877795	4878609	LOC781502	14874	0.23528889
BovineHD0600018463	6	0.000631398	66900340	66889759	66903728	LOC783045	0	0.52026004
BovineHD0600018464	6	0.000631398	66905294	66889759	66903728	LOC783045	1566	0.52026004
BovineHD0600030754	6	0.000726491	109103808	108969761	109535426	LOC618257	0	0.34737772
BTB-01468045	6	0.000873384	4890621	4877795	4878609	LOC781502	12012	0.23663504
BovineHD0800024082	8	0.000269499	80798022	80695448	80787550	LOC507997	10472	0.26655872
BovineHD0800030808	8	0.000326968	103619063	103229817	103621036	MGC142400	0	0.33400897
BovineHD4100007083	8	0.000704729	101183356	101412889	101597077	LOC508100	229533	0.29791281
BovineHD0800022737	8	0.000947254	75877341	76046898	76060032	LOC786296	169557	0.27020818
BovineHD0800022738	8	0.000947254	75879724	76046898	76060032	LOC786296	167174	0.27020818
BovineHD0900021870	9	0.000333714	78450681	78466302	78648155	LOC535975	15621	0.55899512
BovineHD0900021873	9	0.000333714	78460717	78466302	78648155	LOC535975	5585	0.55899512
BovineHD0900021519	9	0.000683699	77116439	77299958	77310361	LOC784274	183519	0.47329351
BovineHD0900009688	9	0.000710144	35336469	35162428	35359069	LOC537689	0	0.46525829
BovineHD1000005571	10	0.00019783	16685022	16672316	16776886	LOC508410	0	0.31028475
BovineHD1000005638	10	0.00019783	16834411	16791129	16837051	MGC139427	0	0.51659603
BovineHD1000002260	10	0.000350639	7207952	7128443	7226417	LOC539768	0	1.23652675
Hapmap39767-BTA-63294	10	0.00037571	30654569	30600612	30803314	LOC617063	0	0.38122442
BovineHD1000029182	10	0.000619578	100772117	94585861	94643745	LOC533428	6128372	0.41819838
BovineHD1000023140	10	0.00076399	81131563	81137445	81159399	LOC617628	5882	0.37735876
BovineHD1000023141	10	0.00076399	81132594	81137445	81159399	LOC617628	4851	0.37735876
BovineHD1000023142	10	0.00076399	81133702	81137445	81159399	LOC617628	3743	0.37735876
BovineHD1000008624	10	0.000954955	26468761	26451800	26722858	HH114	0	0.4116412
BovineHD1000008650	10	0.000954955	26519045	26451800	26722858	HH114	0	0.4116412
BovineHD1000009301	10	0.000954955	28413506	28387367	28468041	LOC533125	0	0.32948674
BovineHD1000009304	10	0.000954955	28418396	28387367	28468041	LOC533125	0	0.32948674
BovineHD1100031338	11	0.000404039	92286220	92208323	92425849	PBX3	0	0.3819536
BovineHD1100027434	11	0.000490874	94182089	94165415	94198257	LOC515820	0	0.50452195
BovineHD1100022797	11	0.000512469	79514927	79212034	79212441	LOC788469	302486	0.31672341

BovineHD1100011491	11	0.000737326	38860380	37968698	38191537	LOC525800	668843	0.28313536
BovineHD1200018816	12	0.000424672	68721674	68443285	68494734	LOC538566	226940	0.39241576
BovineHD1200004537	12	0.000751952	15247033	15244333	15357275	LOC508840	0	0.34226314
BovineHD1200007215	12	0.000782888	24070715	24101505	24233430	LOC533890	30790	0.23182592
ARS-BFGL-NGS-36799	12	0.000913098	79520461	77618072	77618991	LOC787906	1901470	0.37091713
BovineHD1300002685	13	0.000702645	9970486	9921052	9979013	LOC526505	0	0.29914535
BovineHD4100011476	14	0.000340333	38171740	38227426	38386079	LOC511188	55686	0.36247679
BovineHD1400013624	14	0.000472124	48104263	48079258	48176545	EIF3S3	0	0.30824796
BovineHD1400014338	14	0.000497694	50489929	50445561	50452815	LOC781182	37114	0.26866419
BovineHD1400014341	14	0.000497694	50493823	50445561	50452815	LOC781182	41008	0.26866419
BovineHD1400014343	14	0.000497694	50497256	50445561	50452815	LOC781182	44441	0.26849354
BovineHD1400022883	14	0.000570681	80933921	80741670	81112887	LOC617927	0	0.35530945
ARS-BFGL-NGS-1112	14	0.000615325	54947391	55293517	55299467	LOC782496	346126	0.32763817
BovineHD1400015417	14	0.000615325	54972951	55293517	55299467	LOC782496	320566	0.32811732
BovineHD1400005806	14	0.000631398	20350148	20360393	20407780	ATP6V1H	10245	1.38370339
BovineHD1500025112	15	0.000110123	30464452	30006009	30571447	LOC534321	0	0.23577422
BovineHD1500008148	15	0.000376049	30555737	30006009	30571447	LOC534321	0	0.2117454
BovineHD1500012964	15	0.00043358	45961747	45963749	45979275	LOC511036	2002	0.28426431
BovineHD1500008192	15	0.000499131	30675586	30703845	30753373	LOC784497	28259	0.20571471
BovineHD1500003179	15	0.000631398	12498678	12637125	12637418	LOC782283	138447	0.17360285
BovineHD1500019998	15	0.00082709	69258036	69419238	69447810	API5	161202	0.22216474
BovineHD1600023671	16	0.000603411	80981424	72823891	72834138	LOC509797	8147286	0.21033945
BTB-02009238	17	4.56E-05	918620	1049369	1405008	LOC616468	130749	0.48671964
BovineHD1700000459	17	0.000138671	2065041	2131651	2135998	LOC783481	66610	0.40856661
BTB-01990000	17	0.000228236	3500450	3641911	3650486	SFRP2	141461	0.39615297
BovineHD4100012800	17	0.000325901	1958246	2131651	2135998	LOC783481	173405	0.33774253
BovineHD1700000417	17	0.000337226	1808282	1645974	1650185	NPY2R	158097	0.3751305
BovineHD1700000436	17	0.000359047	1928510	2131651	2135998	LOC783481	203141	0.32729816
BovineHD1700000826	17	0.000360119	3493111	3641911	3650486	SFRP2	148800	0.34545076
BovineHD1700000440	17	0.000381151	1945939	2131651	2135998	LOC783481	185712	0.33028105
BovineHD1700001861	17	0.000389319	6479238	6624466	6673982	LOC510523	145228	0.39676254
BovineHD1700001862	17	0.000389319	6484864	6624466	6673982	LOC510523	139602	0.39817571

BovineHD1700000480	17	0.000472282	2162163	2131651	2135998	LOC783481	26165	0.37435383
BovineHD1700000828	17	0.000482351	3503264	3641911	3650486	SFRP2	138647	0.3352939
BovineHD1700000437	17	0.000493478	1938491	2131651	2135998	LOC783481	193160	0.31606679
BovineHD1700000438	17	0.000493478	1942284	2131651	2135998	LOC783481	189367	0.31606679
BovineHD1700000838	17	0.000550955	3570034	3641911	3650486	SFRP2	71877	0.32510132
BovineHD1700000929	17	0.000808965	3923694	3809376	3979377	LOC505156	0	0.28092928
BovineHD1700000867	17	0.000985681	3656789	3641911	3650486	SFRP2	6303	0.34373579
BovineHD1800007657	18	0.000469956	24828404	24677620	24857607	GNAO1	0	0.27716084
Hapmap50690-BTA-43010	18	0.000534397	33447591	33304374	33489759	LOC534015	0	0.28695125
BovineHD1800015971	18	0.000714334	54463089	54476349	54489358	LOC618283	13260	0.23237778
BovineHD1800006939	18	0.000945079	22601714	22533379	22616608	LOC533968	0	0.25100773
BovineHD1900002935	19	0.000359983	10769399	10791768	10819370	LOC615857	22369	0.33481895
BovineHD1900018660	19	0.000740304	9006976	8974082	9194966	LOC537257	0	0.32631358
BovineHD1900004151	19	0.000897328	15378713	15371145	15745690	MGC137743	0	0.24876167
BovineHD1900015991	19	0.000925217	56554454	56555450	56562684	LOC525463	996	0.29488548
BovineHD2000003535	20	7.17E-06	11089453	11002687	11017134	CD180	72319	0.72924832
BovineHD2000003316	20	1.33E-05	10500175	10503094	10550535	OCLN	2919	0.81077297
BovineHD2000020358	20	1.45E-05	69755152	68339491	68341793	LOC619163	1413359	0.52441889
BovineHD2000003303	20	3.59E-05	10462408	10476144	10497539	MGC133741	13736	0.72010269
BovineHD2000003308	20	3.59E-05	10486199	10476144	10497539	MGC133741	0	0.72010269
BovineHD2000001686	20	5.85E-05	5390176	5413657	5439737	GABRP	23481	0.52534235
BovineHD2000001695	20	5.85E-05	5418160	5413657	5439737	GABRP	0	0.52534235
BovineHD2000003531	20	9.81E-05	11083847	11002687	11017134	CD180	66713	0.66373206
BovineHD2000000653	20	0.000101377	1876553	1900624	1909963	LOC781299	24071	0.48340247
BovineHD2000003558	20	0.000105243	11157742	11002687	11017134	CD180	140608	0.45719405
BovineHD2000003685	20	0.000113173	11428343	11621160	11622355	LOC783613	192817	0.50710441
BovineHD2000001689	20	0.000144961	5395165	5413657	5439737	GABRP	18492	0.43718369
BovineHD2000003542	20	0.000151284	11110154	11002687	11017134	CD180	93020	0.43967428
BovineHD2000012369	20	0.000165699	43368589	43349315	43370051	LOC539509	0	0.42931236
BovineHD2000004874	20	0.000169182	16235118	15126146	16793347	LOC539556	0	0.35263904
BovineHD2000004891	20	0.000169182	16273471	15126146	16793347	LOC539556	0	0.35263904
BovineHD2000003569	20	0.000183036	11180923	11002687	11017134	CD180	163789	0.51413072

BovineHD2000002810	20	0.000202689	8946187	8796034	8838048	LOC527467	108139	0.41695926
BovineHD2000003075	20	0.000213877	9730764	9746387	9778197	PTCD2	15623	0.35814046
BovineHD2000003295	20	0.00022068	10413117	10382526	10410258	SMN1	2859	0.55389462
BovineHD2000003357	20	0.000240435	10580238	10594087	10635313	LOC541110	13849	0.40684168
BovineHD2000000659	20	0.000252299	1882638	1900624	1909963	LOC781299	17986	0.38925014
BovineHD2000002756	20	0.000287564	8703472	8721996	8784387	LOC519307	18524	0.43917096
ARS-BFGL-NGS-77784	20	0.000322267	66862841	67394965	67428583	LOC527659	532124	0.58297347
BovineHD2000002556	20	0.00035258	8018916	7718973	7790754	MGC139744	228162	0.35437335
BovineHD2000011971	20	0.000392638	41709705	41698936	41699573	LOC504862	10132	0.31168918
BovineHD2000003666	20	0.000393656	11383301	11621160	11622355	LOC783613	237859	0.40767623
BovineHD2000020111	20	0.000406423	69057382	68339491	68341793	LOC619163	715589	0.42598636
BovineHD2000020361	20	0.000466827	69768129	68339491	68341793	LOC619163	1426336	0.29834666
BovineHD4100014908	20	0.000466827	69772518	68339491	68341793	LOC619163	1430725	0.30754755
BovineHD2000013477	20	0.00048592	48216957	47907148	47907854	LOC783643	309103	0.85530092
BovineHD2000003294	20	0.00049694	10407140	10382526	10410258	SMN1	0	0.57585372
BovineHD2000003306	20	0.00049694	10472213	10476144	10497539	MGC133741	3931	0.57585372
BovineHD2000004877	20	0.000505927	16237996	15126146	16793347	LOC539556	0	0.30454493
BovineHD2000004900	20	0.000505927	16285451	15126146	16793347	LOC539556	0	0.30454493
BovineHD2000004903	20	0.000505927	16292260	15126146	16793347	LOC539556	0	0.30454493
BovineHD2000004910	20	0.000505927	16306497	15126146	16793347	LOC539556	0	0.30454493
BovineHD2000004912	20	0.000505927	16308101	15126146	16793347	LOC539556	0	0.30454493
BovineHD2000004991	20	0.000505927	16466445	15126146	16793347	LOC539556	0	0.30936915
BovineHD2000004992	20	0.000505927	16478171	16470910	16475722	LOC784699	2449	0.30936915
BovineHD2000005003	20	0.000505927	16495702	15126146	16793347	LOC539556	0	0.30936915
BovineHD2000005004	20	0.000505927	16497740	15126146	16793347	LOC539556	0	0.30936915
BovineHD2000005018	20	0.000505927	16545907	15126146	16793347	LOC539556	0	0.30936915
BovineHD2000004882	20	0.000513908	16248353	15126146	16793347	LOC539556	0	0.30221378
ARS-BFGL-NGS-60738	20	0.000515427	12752857	12749867	12796358	LOC787097	0	0.39005404
BovineHD2000003646	20	0.000524165	11342271	11621160	11622355	LOC783613	278889	0.35973751
BovineHD2000003647	20	0.000524165	11343307	11621160	11622355	LOC783613	277853	0.3551355
BovineHD2000003501	20	0.000559617	10989293	10762638	10990083	LOC529061	0	0.34919478
BovineHD2000003320	20	0.000615908	10510212	10503094	10550535	OCLN	0	0.43644268

BovineHD2000001678	20	0.000644879	5360905	5321886	5334114	MGC140092	26791	0.34390988
BovineHD2000012396	20	0.000673649	43494942	43470457	43532818	LOC525869	0	0.28650036
BovineHD2000004603	20	0.000686997	15230293	15126146	16793347	LOC539556	0	0.30027034
BovineHD2000002009	20	0.000693739	6409306	6463181	6463549	LOC783742	53875	0.41351893
BovineHD2000020369	20	0.000704119	69791421	68339491	68341793	LOC619163	1449628	0.27944577
BovineHD2000002702	20	0.000711838	8430484	8494755	8500677	MSX2	64271	0.35065292
BovineHD2000013071	20	0.000733988	46227077	46405477	46405632	LOC786028	178400	0.29354625
BovineHD2000003181	20	0.000892212	10012090	9892591	9985887	MAP1B	26203	0.32810096
BovineHD2000003563	20	0.000920736	11170115	11002687	11017134	CD180	152981	0.36427615
BovineHD2000013521	20	0.000924332	48516564	48602277	48692915	LOC781080	85713	0.30345551
BovineHD2000002552	20	0.000928106	8003809	7718973	7790754	MGC139744	213055	0.31055464
ARS-BFGL-NGS-58314	20	0.000940476	10871544	10762638	10990083	LOC529061	0	0.38082767
BovineHD2000004232	20	0.000964994	13179181	13146497	13266025	LOC511108	0	0.27697677
BovineHD2000004233	20	0.000964994	13182785	13146497	13266025	LOC511108	0	0.27697677
BovineHD2000004919	20	0.000977061	16322498	15126146	16793347	LOC539556	0	0.2840821
BovineHD2100001044	21	6.13E-05	5654518	5647387	5687565	LOC507093	0	0.49838512
BovineHD2100000990	21	8.41E-05	5493691	5226496	5458139	LOC789946	35552	0.42861557
BovineHD4100014935	21	8.41E-05	5499262	5226496	5458139	LOC789946	41123	0.42861557
BovineHD2100001007	21	0.000107849	5535667	5226496	5458139	LOC789946	77528	0.40786871
BovineHD2100001019	21	0.000158105	5585532	5647387	5687565	LOC507093	61855	0.42516157
BovineHD2100001020	21	0.000158105	5588795	5647387	5687565	LOC507093	58592	0.42516157
BovineHD2100001032	21	0.000158105	5624513	5647387	5687565	LOC507093	22874	0.43213285
BovineHD2100001033	21	0.000158105	5625257	5647387	5687565	LOC507093	22130	0.43213285
BovineHD2100001035	21	0.000158105	5629989	5647387	5687565	LOC507093	17398	0.43213285
BovineHD2100001042	21	0.000158105	5650628	5647387	5687565	LOC507093	0	0.43909629
BovineHD2100001046	21	0.000158105	5656349	5647387	5687565	LOC507093	0	0.43909629
BovineHD2100001180	21	0.000246691	6015796	5940934	6030652	CHSY1	0	0.36867736
BovineHD2100001182	21	0.000246691	6023549	5940934	6030652	CHSY1	0	0.36867736
BovineHD2100001191	21	0.000246691	6038824	6061852	6070994	MGC137429	23028	0.36867736
BovineHD2200014891	22	2.78E-05	52426809	52457344	52458963	LOC788085	30535	0.55362262
BovineHD2200014919	22	2.90E-05	52524448	52544750	52545227	LOC614114	20302	0.55142876
BovineHD2200014912	22	0.000120222	52473934	52457344	52458963	LOC788085	14971	0.45161359

BovineHD2200000952	22	0.000271161	3557386	3531543	3727195	LOC614548	0	0.40028462
BovineHD2200014876	22	0.000374382	52398798	52183488	52340582	MGC142412	58216	0.35833075
ARS-BFGL-NGS-43481	22	0.000502852	57930390	57912718	57926714	LOC507939	3676	0.34614188
BovineHD2200017368	22	0.000749227	59828585	59803391	59852680	LOC532521	0	0.33416194
BovineHD2200013110	22	0.000913908	45326875	44995625	45282086	LOC519644	44789	0.35044706
BovineHD2300001341	23	0.000100249	5584389	5474689	5585785	LOC618855	0	0.42876264
BovineHD2300000776	23	0.000663904	3588320	3587189	3684384	LOC790110	0	0.25881084
BovineHD2300000777	23	0.000663904	3589476	3587189	3684384	LOC790110	0	0.25881084
BovineHD2300009607	23	0.00075485	32952316	32896314	33039963	LOC541284	0	0.23546628
BovineHD2400000873	24	3.75E-06	3255562	3239715	3244309	LOC521016	11253	0.5145984
BovineHD2400000876	24	3.75E-06	3262266	3239715	3244309	LOC521016	17957	0.5145984
BovineHD2400000883	24	6.54E-06	3281562	3239715	3244309	LOC521016	37253	0.49749457
BovineHD2400000886	24	6.54E-06	3302265	3239715	3244309	LOC521016	57956	0.49379264
ARS-BFGL-NGS-2531	24	5.81E-05	6183550	6015554	6254293	LOC790283	0	0.38968642
BovineHD2400000884	24	0.000171206	3292241	3239715	3244309	LOC521016	47932	0.34110758
BovineHD2400001238	24	0.000424767	4557059	4196312	4197792	LOC790254	359267	0.38800988
BovineHD2400006511	24	0.00058046	23848246	23903763	23976801	LOC507330	55517	0.34321866
BovineHD2400006508	24	0.000607853	23835665	23903763	23976801	LOC507330	68098	0.32200992
BovineHD2400017460	24	0.000777015	60413692	59897148	60006017	LOC525095	407675	0.31067791
BovineHD2400000921	24	0.000792797	3423617	3239715	3244309	LOC521016	179308	0.32973748
BovineHD2400018086	24	0.000882375	62205149	59897148	60006017	LOC525095	2199132	0.344237
BovineHD2400000657	24	0.000954347	2494457	2314638	2315434	LOC790035	179023	0.33396854
BovineHD2500011602	25	0.000647696	41106700	40897073	41496081	LOC516333	0	0.28937045
BovineHD2500010718	25	0.000758467	38464228	38451075	38473460	LOC538021	0	0.30647207
BovineHD2600009937	26	0.00075179	36372215	36143857	36406716	GPRK5	0	0.25295395
Hapmap44556-BTA-66734	27	0.000370635	22839206	22890021	22931823	LOC533821	50815	0.44710828
BovineHD2700005738	27	0.000591605	20164451	20360948	20618283	LOC784004	196497	0.2952349
BovineHD2700005994	27	0.000631375	21036240	21163084	21163921	LOC784180	126844	0.27801391
BovineHD2700005733	27	0.000710631	20140892	20360948	20618283	LOC784004	220056	0.28383906
BovineHD2700005925	27	0.000807663	20828955	20360948	20618283	LOC784004	210672	0.28352069
BovineHD2700003135	27	0.000821496	10486904	10972158	11044957	LOC784124	485254	0.22951597
BovineHD2700005734	27	0.000848824	20142691	20360948	20618283	LOC784004	218257	0.27588243

BTB-00982394	28	2.53E-05	24007807	23906631	24062648	LOC613849	0	1.83580866
BovineHD2800002008	28	0.000881873	6747420	6730542	6738364	LOC782106	9056	0.22813555
ARS-BFGL-NGS-105175	30	2.53E-05	71351341	71167036	71170431	LOC523373	180910	0.26267465
BovineHD3000040166	30	0.000155305	139521609	0	0	0	0	0.20586443
BovineHD3000040251	30	0.000155305	139803080	0	0	0	0	0.20586443
BovineHD3000046712	30	0.000159023	67406152	67507986	67513052	LOC785265	101834	0.21077926
BovineHD3000046047	30	0.000842552	12182908	12876359	12882264	LOC541035	693451	0.16934995
BovineHD3000034585	30	0.000886587	122605732	99863691	99873236	LOC529792	22732496	0.12310371
BovineHD3000034589	30	0.000886587	122616708	99863691	99873236	LOC529792	22743472	0.12310371
BovineHD3000005757	30	0.00095246	17365729	17592723	17602162	LOC530936	226994	0.14386876
BovineHD3000005758	30	0.00095246	17375211	17592723	17602162	LOC530936	217512	0.14386876
