# Supplementary material for: Multi-Trait GWAS and New Candidate Genes Annotation for Growth Curve Parameters in Brahman Cattle
Source: PLoS One. 2015 Oct 7;10(10):e0139906. doi: 10.1371/journal.pone.0139906 (PMC4622042; doi:10.1371/journal.pone.0139906)
Supplement: S3 Table — (PDF) [file pone.0139906.s008.pdf]

S3 Table. Blocks formed by linkage disequilibrium among the 167 significant SNPs for A (mature weight) and 262 significant SNPs for K (maturity rate). %var: additive genetic variance explained by blocks (%).

Trait	Haplotypes	SNPs	Chr	Position	Nearest Gene	%Var
A	Haplotypes 1	2	8	14927629-15022993	MGC133601	1.32
	Haplotypes 2	3	16	76187883-76200952	LOC509797	1.51
	Haplotypes 3	5	19	59614413-59745154	LOC787823	2.37
	Haplotypes 4	2	27	41551568-41618491	LOC784356	0.80
	Haplotypes 5	2	X	65152485-65221973	LOC784159	0.44
K	Haplotypes 1	6	1	29098485-119188091	LOC781270	1.94
	Haplotypes 2	2	2	94542687-94557690	LOC782360	0.49
	Haplotypes 3	2	3	37226889-37411646	LOC787616	0.65
	Haplotypes 4	8	4	18168180-117064985	LOC512582,LOC788066.LOC614377	2.49
	Haplotypes 5	3	5	69831255-69885003	MGC137188	0.97
	Haplotypes 6	2	6	5028589-5065951	LOC781502	0.50
	Haplotypes 7	2	6	49942445-66900340	LOC783045	0.92
	Haplotypes 8	2	11	79514927-94182089	LOC515820	0.82
	Haplotypes 9	2	14	50493823-50497256	LOC781182	0.54
	Haplotypes 10	2	18	24828404-54463089	GNAO1	0.51
	Haplotypes 11	3	20	9778197-10410258	PTCD2,SMN1,MAP1B	1.26
	Haplotypes 12	2	20	10871544-12752857	LOC787097	0.77
	Haplotypes 13	3	20	16308101-16466445	LOC539556	0.90
	Haplotypes 14	4	21	5656349-6038824	CHSY1,MGC137429	1.55
	Haplotypes 15	3	22	3557386-52398798	LOC519644,MGC142412	1.11
	Haplotypes 16	2	22	52473934-52524448	LOC788085,LOC614114	1.00
	Haplotypes 17	2	23	3588320-3589476	LOC790110	0.52
	Haplotypes 18	2	24	23835665-23848246	LOC507330	0.67
	Haplotypes 19	2	27	21036240-22839206	LOC784180	0.73
