# Supplementary material for: Multi-Trait GWAS and New Candidate Genes Annotation for Growth Curve Parameters in Brahman Cattle
Source: PLoS One. 2015 Oct 7;10(10):e0139906. doi: 10.1371/journal.pone.0139906 (PMC4622042; doi:10.1371/journal.pone.0139906)
Supplement: S4 Table — Estimates for growth curve parameters: mature weight (A), scale (b), and maturity rate (K). Goodness of fit measures: LOGLIK, Akaike Information Criterion (AIC), and Bayesian Information Criterion (BIC), using nlme function by R software. (PDF) [file pone.0139906.s009.pdf]

S4 Table. Comparison between four nonlinear growth models (Gompertz, Logistic, Brody and von Bertalanffy) using different covariance matrix structures (diagonal, corAR1, corCAR1, corARMA1. Estimates for growth curve parameters: mature weight (A), scale (b) and maturity rate (K). Goodness of fit measures: LOGLIK, Akaike Information Criterion (AIC) and Bayesian Information Criterion (BIC), using *nlme* function by R software.

Diagonal covariance matrix

		Gompertz	Logistic	Brody	von Bertalanffy
	A	419.90(51.44)	399.85(42.67)	520.32(163.72)	373.86(36.26)
Parameters	b	2.00(0.12)	4.85(0.64)	0.92(0.02)	0.75(0.04)
	k	0.13(0.03)	0.20(0.04)	0.06(0.02)	0.08(0.02)
	LOGLIK	-36376.34	-37239.25	-35127.27	-40026.96
Goodness of fit	AIC	72772.69	74498.51	70274.55	80073.91
	BIC	72841.95	74567.77	70343.81	80143.18

Structured covariance matrix (corAR1)

		Gompertz	Logistic	Brody	von Bertalanffy
	A	*	415.75(3.19)	*	370.27(2.83)
Parameters	b	*	4.31(0.26)	*	0.69(5.68e-06)
	k	*	0.17(0.46)	*	0.08(8.87e-07)
	LOGLIK	*	-36541.81	*	-39335.06
Goodness of fit	AIC	*	73105.62	*	78692.12
	BIC	*	73181.81	*	78768.31

Structured covariance matrix (corCAR1)

		Gompertz	Logistic	Brody	von Bertalanffy
	A	409.44(3.05e+01)	394.85 (2.81e+01)	471.24(3.68e+01)	374.291.69e+01)
Parameters	b	1.98(4.24e-05)	4.65 (1.14e-04)	0.91(7.24e-07)	0.73(9.64e-06)
	k	0.13(1.12e-08)	0.19(3.41e-06)	0.06(2.82e-06)	0.08(8.90e-07)
	LOGLIK	-36376.34	-37239.25	-35127.27	-40026.96
Goodness of fit	AIC	72774.69	74500.51	70276.55	80075.91
	BIC	72850.88	74576.7	70352.74	80152.11

Structured covariance matrix (corARMA1)

		Gompertz	Logistic	Brody	von Bertalanffy
Parameters	A	451.65(46.07)	419.27(3.59e+01)	*	366.72(3.10e+01)
	b	1.90(0.14)	4.27(6.36e-01)	*	0.69(4.13e-06)
	k	0.11(0.01)	0.16(3.61e-06)	*	0.08(3.27e-07)
Goodness of fit	LOGLIK	-35704.57	-36352.8	*	-39013.37
	AIC	71433.13	72729.61	*	78050.73
	BIC	71516.25	72812.73	*	78133.85
